# Loss of Gravitropism in Farnesene-Treated *Arabidopsis* Is Due to Microtubule Malformations Related to Hormonal and ROS Unbalance

**DOI:** 10.1371/journal.pone.0160202

**Published:** 2016-08-04

**Authors:** Fabrizio Araniti, Elisa Graña, Urszula Krasuska, Renata Bogatek, Manuel J. Reigosa, Maria Rosa Abenavoli, Adela M. Sánchez-Moreiras

**Affiliations:** 1 Dipartimento di AGRARIA, Università Mediterranea di Reggio Calabria, Facoltà di Agraria – Salita Melissari, Lotto-D, I-89124, Reggio Calabria RC, Italy; 2 Department of Plant Biology and Soil Science, University of Vigo, Campus Lagoas-Marcosende s/n, E-36310, Vigo, Spain; 3 Department of Plant Physiology, Warsaw University of Life Sciences-SGGW, Nowoursynowska 159, 02-776, Warsaw, Poland; Iwate University, JAPAN

## Abstract

Mode of action of farnesene, a volatile sesquiterpene commonly found in the essential oils of several plants, was deeply studied on the model species *Arabidopsis thaliana*. The effects of farnesene on the *Arabidopsis* root morphology were evaluated by different microscopic techniques. As well, microtubules immunolabeling, phytohormone measurements and ROS staining helped us to elucidate the single or multi-modes of action of this sesquiterpene on plant metabolism. Farnesene-treated roots showed a strong growth inhibition and marked modifications on morphology, important tissue alterations, cellular damages and anisotropic growth. Left-handed growth of farnesene-treated roots, reverted by taxol (a known microtubule stabilizer), was related to microtubule condensation and disorganization. As well, the inhibition of primary root growth, lateral root number, lateral root length, and both root hairs length and density could be explained by the strong increment in ethylene production and auxin content detected in farnesene-treated seedlings. Microtubule alteration and hormonal unbalance appear as important components in the mode of action of farnesene and confirm the strong phytotoxic potential of this sesquiterpene.

## Introduction

The increasing occurrence of resistance in synthetic herbicides-treated weeds, first observed by Ryan [1], has attracted attention during the last years as over 300 biotypes of weeds have become resistant to herbicides, with an average occurrence of nine cases per year [2].

Reduced herbicide concentration at the site of action, metabolism of the herbicide to nontoxic species, and loss of affinity for the site of action are among the most frequent causes of herbicide resistance [3]. It is therefore necessary to vary the chemical class and dose of the applied compounds in order to prevent the emergence of herbicide resistance. Unluckily, in the last twenty years, because of national law restrictions or because of the availability of more generic weed-killing products, no herbicides with new modes of action have been introduced in the market [4].

Herbicides with new modes of action are badly needed to manage the evolution of resistance of weeds to existing herbicides, and natural products represent a vast basin of molecules with evolved phytotoxic activity. Moreover, these compounds could be used directly or could be introduced in programs of chemical synthesis as templates for the production of new natural derivatives with new modes of action [5].

As a result of natural selection, plants produce and emit a large variety of volatiles that play important roles as direct or indirect signals in plant-herbivore protection and plant-plant communication, and have multiple biological activities [6, 7]. Terpenoids are the largest group of plant volatiles and the primary constituents of floral and herbivore-induced leaf volatile blends in many plants [8]. Terpenoids are derived from the universal C5 precursor isopentenyl diphosphate and its allylic isomer dimethylallyl diphosphate, which form a plethora of compounds derived from the branched C5 skeleton of isoprene, including volatile monoterpenes (C10) and sesquiterpenes (C15).

The sesquiterpene farnesene is an acyclic semiochemical extensively involved in both plants- and insects- communication [9]. This volatile is considered a potential defense against aphids and an aphid alarm pheromone, being used by the plant as an allomone [10]. Emitted by the Dufour’s gland of andrenid bees [11] and by several genera of ants [12, 13], farnesene plays an important role both as a defensive allomone and a trail pheromone [14].

Although its effects on plant-insect or insect-insect interactions have been broadly demonstrated, little information is available about its role on plant-plant interaction and its phytotoxic potential. Recently, *in vitro* studies showed a strong dose-dependent toxic effect of farnesene on *Arabidopsis thaliana* roots [15] causing root hairs inhibition and a significant deformation of the root (corkscrew-shaped), defined as "handedness" probably due to the anisotropic growth of root cells. This phenomenon, well known in nature, is defined as "left or right handedness" depending on the side of the twist [16] and could interest both shoot and root systems [17]. In a review, Ishida et al. [18] described the phenomenon of the twisted growth and microtubule organization, including that mutants showing fixed direction, left- or right-handedness, and exhibited defects in microtubules arrangement [19, 20]. As previously observed for the microtubule stabilizer taxol and the microtubule disruptor propizamyde, [16] farnesene-treated roots showed unidirectional "left handedness", suggesting microtubule dynamic instability [19].

Although similar effects were observed with colchicine, taxol and oryzalin [21, 22], different mechanisms of action have been proposed for these molecules. Indeed, oryzalin and trifluralin inhibited the polymerization of tubulin into microtubules, whereas the dinitroanilines and phosphoric amides inhibited the formation of both mitotic and cortical microtubules causing the characteristic swollen cellular morphology, the formation of large and irregular nuclei, and the consequently mitotic blockage [23, 24].

Other natural compounds disrupting mitotic activity and affecting microtubule stability were also studied. As reported by Deysson [25] and Vaughn & Vaughn [26], the podophyllotoxin isolated from *Podophyllum peltatum* was able to disrupt mitosis at prometaphase in a similar way to that of colchicine but binding to a different site on the plant tubulin molecule [27]. Vinblastine and vincristine, two alkaloids isolated from plants of the genus *Vinca* sp., induced multipolar division [28]. In a similar way, Oliva et al. [29] found that the lignan etoposide strongly affected root growth inducing abnormal star anaphase chromosomal configurations and altering the formation of the spindle microtubular organization centers. Recently, several authors [22, 30] demonstrated that the terpene citral strongly affected cell division by disrupting mitotic and cortical microtubules and causing a strong alteration on hormonal balance, especially ethylene and indole-3-acetic acid (IAA). These compounds, which act on cell division processes, have prompted the interest for their future use as natural herbicides.

Phytotoxins are known as stress inducers in acceptor plants, and thus can modulate reactive oxygen species (ROS) metabolism [31]. Burst of superoxide anion (O_2_^-^.), and more stable hydrogen peroxide (H_2_O_2_) is commonly observed as a result or/and reaction to different stress factors. Furthermore, together with ROS, reactive nitrogen species (RNS) are known to be involved in plant-plant environmental interactions [32]. Moreover, ROS accumulation in stressed plants can affect plant hormones homeostasis [33, 34], since they act as signaling molecules involved in multiple physiological processes controlled by phytohormones. For example, auxin production can be induced by ROS, and ROS-induced auxin is involved in the regulation of cell elongation [35], or in root gravitropic responses [36]. Moreover, auxin polar transport is controlled by both ROS and RNS [32]. The balance between these three components (auxin/ROS/RNS) is essential for the generation of secondary roots, root hairs and root gravitropism [32]. In addition, an excessive ROS accumulation may broke the hormonal equilibrium [31], causing alterations at both physiological and morphological levels.

Therefore, the present study evaluates the effects of farnesene on root morphology, ultrastructure and microtubules organization of *A*. *thaliana* root tips. The involvement of hormones, such as auxin and ethylene, will be discussed as well in order to understand the underlying mechanism/s responsible for the farnesene induced left asymmetry and strong growth inhibition and to elucidate the single- or multi-modes of action of farnesene. Additionally alteration of ROS and nitrogen oxide (NO) levels in *Arabidopsis* roots treated with this compound will be revealed.

## Materials and Methods

### Root morphology of *Arabidopsis thaliana*

Seeds of *A*. *thaliana* (L.) Heynh. ecotype Columbia (Col-0) were sterilized as described by Araniti et al. [37]. In particular, seeds were rinsed for 3 min in 50% EtOH and 0.5% NaOCl with Triton X-100 at 0.01% and washed for three times in autoclaved ultrapure water. After sterilization, seeds were immersed in 0.1% agar solution at 4°C for 72 h to allow vernalization. Twenty-four seeds were then placed in square Petri dishes (100 x 150 mm) containing plant agar (0.8% w/v) with a mixture of micro- and macro-nutrients (basal salt-medium Murashige-Skoog, Sigma-Aldrich) supplemented with 1% sucrose (pH 6.0). The plates were placed vertically in the growth chamber at 22 ± 2°C, 55% relative humidity, 75 μmol m^-2^s^-1^ light intensity and 8/16 h (light/darkness) photoperiod to encourage geotropic root growth.

Once germinated, 24 seedlings were transferred to a single plate and grown in the same medium containing 0, 50, 100, 200, 400, 800 or 1200 μM farnesene (Sigma-Aldrich) for 14 days. The molecule was dissolved in ethanol and the same amount of solvent (0.1% v/v) was added to the control. Whole root systems were then imaged by scanning (STD 1600, Régent Instruments Inc., Quebec, Canada), and primary root length (PRL), number of lateral roots (NLR) and lateral root length (LRL) were evaluated with a WinRhizo Pro System v. 2002a (Régent Instruments Inc., Quebec, Canada). Root hair length (RHL) and root hair density (RHD) were evaluated as well with an Olympus microscope (SZX9).

### Root gravitropism analyses

To evaluate the root response to gravitropic stimulus, germinated *A*. *thaliana* seeds (cropped as previously described) were immediately transplanted to a new medium containing farnesene (0 and 250 μM). After one week, Petri dishes containing the vertically grown seedlings (treated and untreated) were rotated 90° to gravistimulate the roots. Root curvature was monitored for 96 hours and the effects observed during root reorientation were scored using Image Pro Plus (Media Cybernetics).

### Farnesene and taxol growth bioassays

*Arabidopsis* seedlings, germinated and grown as previously described, were treated with farnesene and/or taxol, both previously dissolved in ethanol (0.1% final volume) and added to the agar medium. The treatments consisted in 250 μM farnesene, 0.25 or 0.5 μM taxol or a combination of both compounds (0.25 μM taxol + 250 μM farnesene, and 0.5 μM taxol + 250 μM farnesene). Ethanol (0.1%) was used also in the control. Four replicates were performed for each treatment. Fourteen days after treatment, root meristems were analyzed with a Nikon SMZ 1500 microscope, and the presence of helical twist and the root angle deviation were evaluated and compared to the control by using the software Image Pro Plus (Media Cybernetics).

### Structural and ultra-structural root studies

Root tips of 40 *A*. *thaliana* seedlings treated with farnesene (250 μM) for 7 or 14 days were cut and immediately dipped in 0.1 M cacodylate buffer (pH 7.2) containing glutaraldehyde fixative (5%) for 4 h and then washed 3 times (4 h each) with 0.1 M cacodylate buffer (pH 7.2). Samples were immersed for 3 h in 0.1 M cacodylate buffer with osmium tetroxide (2%) and then in 10% acetone with 2% uranyl acetate for 1 h. Sample dehydration was performed with successively different acetone dilutions: 50% acetone (2 × 30 min), 75% acetone (2 × 1 h), 80% acetone (2 × 1 h), 95% acetone (2 × 1 h), and 100% acetone (2 × 2 h). Then, each sample was embedded in Spurr’s resin as follows: Spurr: acetone (1:3 v/v) (3 × 2 h), Spurr: acetone (1:1 v/v) (3 × 2 h), Spurr: acetone (3:1 v/v) (2 × 2 h plus 1 × 3 h). All steps of fixation, dehydration and Spurr’s resin impregnation were performed at 4°C. Once the included material was embedded in 100% resin for 3 h, it was left overnight at room temperature and included again in 100% resin (2 × 3 h). Samples were then placed in molds with pure resin to allow polymerization at 60°C for 2–3 days. Semi-thin (0.7 μm) and ultrathin sections (50–70 nm) were prepared for light and electron microscopy, respectively. The contrast of ultrathin sections was enhanced in uranyl (2%) for 30 min and lead citrate for 12 min (according to Reynolds, 1963). The ultrathin sections were assembled on copper grids of 100 and 200 mesh and examined by transmission electron microscopy (TEM) using a JEOL JEM-1010 transmission electron microscope (at 100 kV) (Peabody, MA, USA) equipped with a CCD Orius-Digital Montage Plug-in camera (Gatan Inc., Gatan, CA, USA) and Gatan Digital Micrograph software (Gatan Inc.).

The semithin sections were examined using a Nikon Eclipse 800 microscope with a Sight DS-U2 Nikon digital camera and a NIS-Elements D 2.30 SP1 software.

### Microtubules visualization by electron microscopy (TEM)

Microtubules visualization was conducted according to Holzinger et al. [38]. Approximately 50 root tips from 7 or 14-days old *A*. *thaliana* seedlings were cut and fixed in 2.5% glutaraldehyde prepared in 50 mM sodium cacodylate buffer, pH 7.0 at 20°C for 2 h. Then, samples were washed and post-fixed in 1% OsO_4_ in the same buffer at 4°C for 12 h; dehydrated in increasing concentrations of ethanol and, finally, infiltrated in Spurr’s resin and polymerized at 70°C for 8 h. Ultrathin sections were analyzed with the JEOL JEM-1010 transmission electron microscope (at 100 kV) with a CCD Orius-Digital Montage Plug-in camera and Gatan Digital Micrograph software.

### Microtubules immunolabeling

The microtubules immunolabeling was carried out according to the method of Holzinger et al. [39] with some modifications. *A*. *thaliana* roots treated with 0 or 250 μM farnesene and with 250 μM farnesene + 0.5 μM taxol for 7 or 14 days were harvested and fixed at room temperature for 45 min in PEM buffer (50 mM PIPES, 2 mM EGTA, 2 mM MnSO4; pH 7.2) containing 0.5% glutaraldehyde, 1.5% formaldehyde and 0.1% Triton X-100. Then, roots were washed in the same buffer for 20 min and once again in PEM buffer. After chopping, the samples were digested with cellulase and pectoliase Y-23 (1%) (Sigma-Aldrich) in PEM (pH 5.5), at room temperature for 30 min. After digestion, the samples were washed in PEM (pH 7.2) and incubated in methanol at -20°C for 10 min. They were then washed with phosphate buffer saline (PBS) (0.8% NaCl, 0.02% KCl, 0.02% K_2_HPO_4_, 8.1 mM Na_2_HPO_4_, pH 7.4) and incubated with 1 mg mL^-1^ sodium borate in PBS for 20 min. The samples were further washed with PBS and incubated with 1% BSA and 50 mM glycine in PBS. After a final washing in PBS, the samples were incubated overnight with the primary antibody (anti-α tubulin B 5-1-2, Sigma-Aldrich, 1:1000 in PBS) at 4°C, which was removed by three consecutive washes in PBS. The samples were then incubated with the secondary antibody (Alexa 488-conjugated goat anti-mouse IgG, Sigma-Aldrich, 1:200 in PBS) at 37°C for 3 h. Finally, they were mounted in Citifluor AF1 antifade agent. Visualization was performed using a Leica TCS SP5 confocal microscope (Wetzlar, Germany) with a 63X oil immersion objective and a 496 nm excitation wavelength (argon laser); and photographed with a LAS-AF software.

### Auxin quantification

Indoleacetic acid (IAA) quantification was determined in *A*. *thaliana* seedlings treated with 250 μM farnesene for 7 or 14 days. The samples were analyzed by enzyme-linked immunosorbent assay (ELISA) as described by Elsorra et al. [40] with some modifications. Roots and shoots of *A*. *thaliana* were separated and homogenized in 80% (v/v) methanol with 20 mg L-1 of butylated hydroxytoluene (BHT). The homogenized samples were then incubated at -80°C for 24 h and centrifuged at 9500 × g at 4°C for 15 min. Once the supernatant was collected, the remain pellet was extracted with 80% (v/v) methanol and centrifuged again at 9500 × g at 4°C for 15 min, and both supernatants were then mixed for analysis.

The standard IAA solution was prepared in 80% MeOH and diluted at the desired concentrations. The IAA standards and the samples were methylated by 2.0 M trimethylsilyldiazomethane solubilized in hexane for 30 min at room temperature. The reaction was stopped with 0.05 N acetic acid. After evaporation of methanol, samples were re-suspended in PBS (pH 6.9). Methylated IAA (Sigma-Aldrich) standard was used as a positive control.

ELISA plates were filled with rabbit anti-mouse antibodies IgG (RAMIG; Sigma-Aldrich), (25 μg mL-1 prepared in 0.05 M NaHCO3, pH 9.6) and incubated at 4°C overnight. After antibodies removing, the wells were filled with 200 μL monoclonal anti-auxin (1 μg mL-1) (Sigma-Aldrich) and incubated at 4°C overnight. The plates were then carefully rinsed with ultrapure water, and the samples, the IAA standards and the positive control were incubated with 100 μL TBS buffer per well at 4°C for 1 h. After incubation, the plates were filled with 50 μL IAA tracer [Agrisera; 3 μL in 5 mL TBS buffer with 0.1% (w/v) gelatin], incubated at 4°C for 3 h, and rinsed twice with ultrapure water. For enzymatic reaction, 200 μL p-nitrophenylphosphate disodium solution (1 mg mL-1) (Sigma-Aldrich) was added to each plate before incubation at 37°C for 1 h. After stopping the reaction with 5 M KOH, IAA concentration was measured at 405 nm with referential wave of 605 nm using a Dynatech Microplate Reader MR 5000. The experiments were performed by triplicate and the results presented as percentage of the control.

### Ethylene emission measurement

Ethylene was quantified with a gas chromatograph (Hewlett Packard 5890 Series II) equipped with a flame ionization detector (FID) and a stainless steel column (6 fit x 1.8 inch x 2.1 mm) packed with Poropack Q. Roots and cotyledons were separately transferred to 8 mL glass vials containing 0.5 mL distilled water or 0.5 mL 1 mM ACC 1-aminocyclopropane-1-carboxylic acid (ACC) water solution to obtain the maximal ethylene emission [30]. In order to remove the ethylene produced after seedlings cutting, the vials were left open at room temperature for 30 min and successively sealed and incubated at 30°C for 3 h in dark conditions. After incubation, 1 mL of the headspace gas sample was injected in the gas chromatographer and the ethylene emission was detected. Detector and injector were set at 150 and 120°C, respectively. All determinations were performed in triplicate and the results were presented as percentage of the control.

### In situ semi-quantitative detection of H_2_O_2_ and O_2_^−^

After 250 μM farnesene treatment for 7 and 14 days, roots tips from *A*. *thaliana* seedlings (5 per treatment) were cut and immediately immersed in distilled water. They were then vacuum infiltrated for 5 min with a 0.65 mg mL^−1^ solution of sodium azide (NaN_3_) in potassium phosphate buffer (pH 7.8) containing 0.1% (w/v) nitroblue tetrazolium (NBT) for superoxide (O_2_^-^) detection or in acidified water (pH 3.8) containing 3,3-diaminobenzidine (DAB) for hydrogen peroxide (H_2_O_2_) localization [41]. After infiltration, roots were left in the same buffer in darkness for 20 min and then illuminated until the appearance of the characteristic reddish brown or dark blue color for DAB or NBT staining, respectively.

### Determination of NO emission

Nitrogen oxide (NO) emission from *A*. *thaliana* roots treated with 250 μM farnesene for 7 or 14 days was measured using 4,5-diaminofluorescein diacetate (DAF-FM DA; Invitrogen), a specific NO fluorescent probe. Isolated roots were rinsed in 10 mM HEPES–KOH (pH 7.4) for 15 min. Successively, four roots were transferred to a test tube containing 100 μL of HEPES-KOH (pH 7.4) with 20 μM of DAF-FM DA. The tubes were placed in darkness on a continuous orbital shaker for 1 h at room temperature. After incubation roots were washed in buffer for three times and transferred to a cuvette containing 0.8 mL HEPES–KOH (pH 7.4). Fluorescence was recorded for 1800 s with a fluorescence spectrophotometer Hitachi F-2500 (excitation 495 nm and emission 515 nm). All measurements were repeated three times, and fluorescence emission was normalized per mg of fresh weight and expressed in arbitrary units. One unit was defined as fluorescence emission obtained for the control plants.

### Statistical analysis

All the experiments were carried out with a completely randomized design. Data were evaluated for normality with the Kolmogorov-Smirnov test and for homogeneity of variances with the Levene’s test. The statistical significance of differences among group means was estimated by analysis of variance followed by Least Significant Difference tests (LSD) in case of homoscedastic data, and by Tamhane’s T2 test in case of heteroscedastic data (P ≤ 0.05). Data regarding ultrastructure parameters and gravitropism bioassay were evaluated through the t-test with P ≤ 0.05. Statistical analysis were performed using the software SPSS, version 11.5 (SPSS, Chicago) and Oriana software (Kovach Computing Services) for root gravitropism and taxol experiments.

## Results

### Root morphology of *A*. *thaliana*

*In vitro* bioassays are a rapid and cost effective method which allow to get, in a relatively short period, a panoramic of the macroscopic effects caused by the molecule, as well as information regarding the potential target and mode of action, allowing to better plan the next steps of the research.

**Fig 1:**
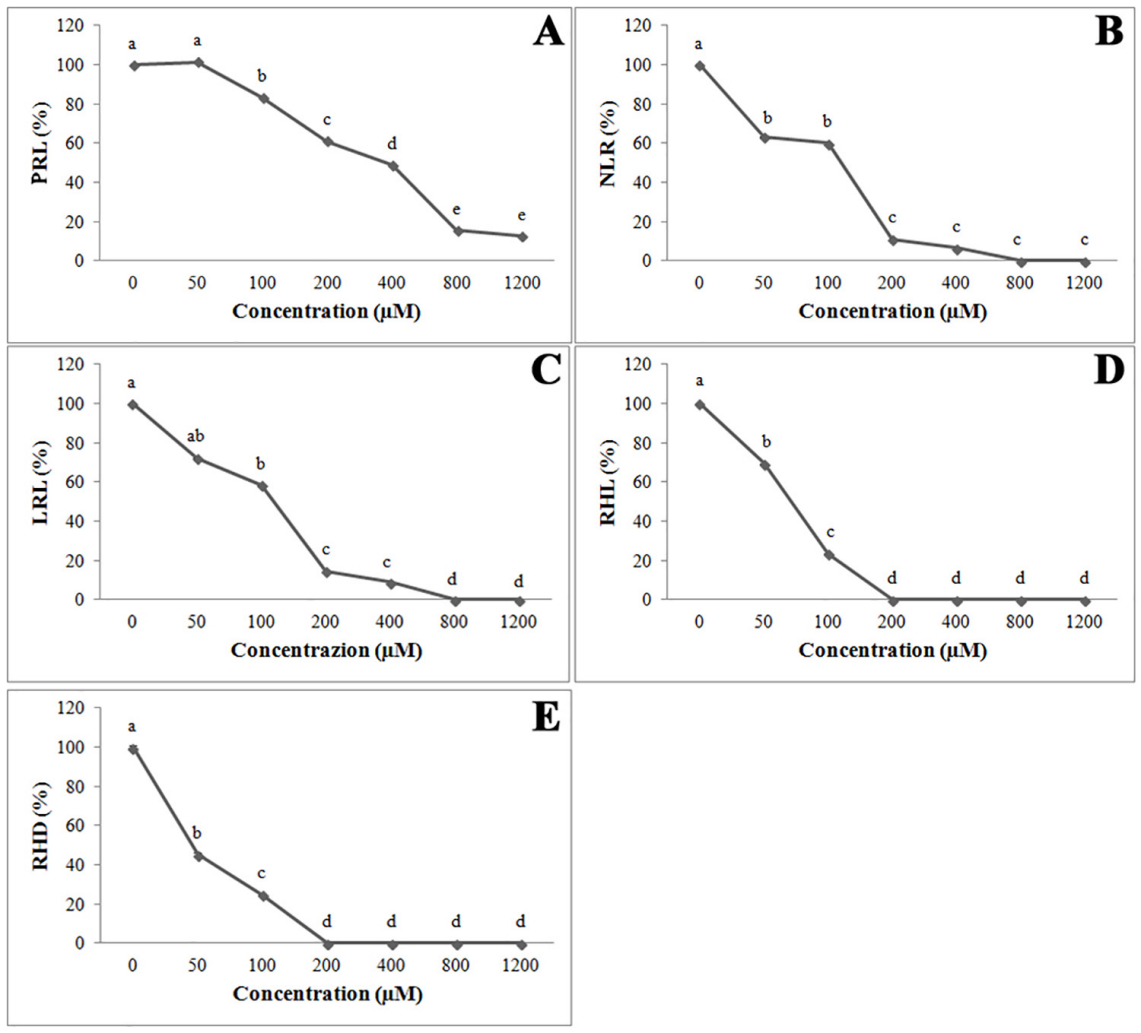
Primary root length, number of lateral roots, lateral roots length, root hair length, and root hair density. A) Dose-response curves of PRL (primary root length), B) NLR (number of lateral roots), C) LRL (lateral roots length), D) RHL (root hair length), and E) RHD (root hair density) of 250 μM farnesene-treated *Arabidopsis* seedlings for 14 days. Data are expressed as percentage of the control. Different letters indicate significant differences among treatments at *P* ≤ 0.05. N = 5.

In the present study the results obtained from the study of root morphology confirmed the dose-dependent strong inhibition induced by farnesene on primary root length of *A*. *thaliana* (Fig 1A), as previously demonstrated by Araniti et al. [15]. Primary root length (PRL) was inhibited by 40% compared to the control when treated with 200 μM farnesene, reaching more than 85% inhibition at higher concentrations (800 and 1200 μM) (Fig 1A). The IC_50_ and IC_80_ values (323 μM and 780 μM farnesene, respectively; [15]) were also confirmed. Moreover, farnesene strongly affected all root morphological parameters, with 200 μM being the threshold concentration for complete inhibition (Fig 1B–1E). The lateral roots number (NLR) was already significantly inhibited at 50 μM with a 37% reduction compared to the control, reaching the 100% inhibition at the highest concentrations (800 and 1200 μM; Fig 1B). The lateral roots length (LRL) showed the same trend, being significantly inhibited (42% of the control) at 100 μM farnesene and completely inhibited at the highest concentrations (Fig 1C). Moreover, a marked inhibition in root hair density (RHD) and length (RHL) was already detected at 50 μM, and was complete at 200 μM (Fig 1D and 1E).

**Fig 2:**
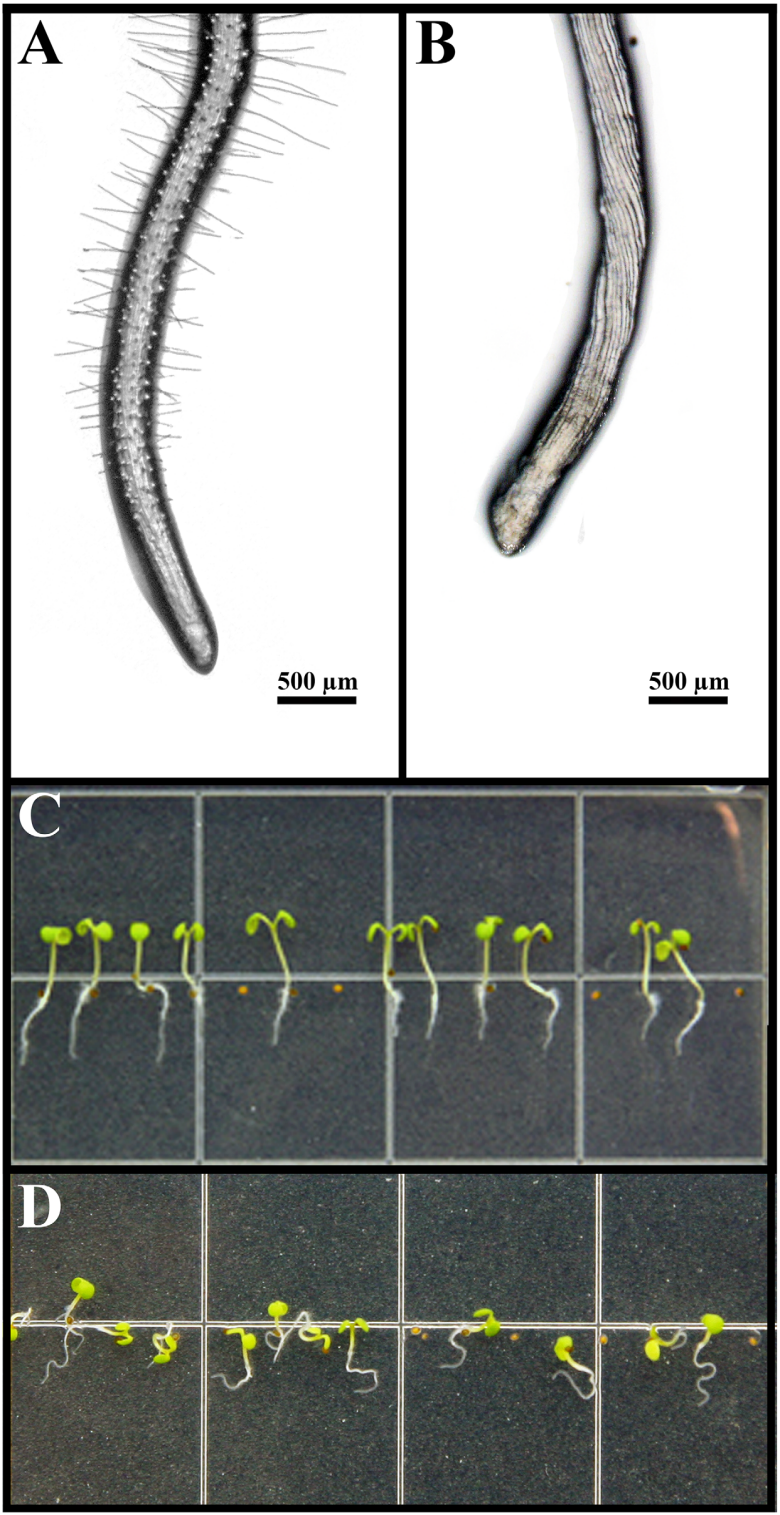
Morphology of *Arabidopsis* root tips. *Arabidopsis* root tips grown on agar medium (A) Control root tip with root hairs (B) Root tips treated with 250 μM farnesene. Note the absence of root hairs and the anticlockwise torsion of the cell lines (left-handed). On the bottom is reported a panoramic of young *Arabidopsis* seedlings grown on agar medium untreated (C) and treated with 250 μM farnesene (D). Note the anisotropic growth of the root and the apparent loss of gravitropic response.

These morphological alterations were easily identified with the aid of a stereo microscope (Fig 2). While untreated *A*. *thaliana* roots showed symmetric rows of cells in well-characterized elongation and differentiation zones, as well as root hair presence (Fig 2A), farnesene-treated seedlings showed bold roots and strong tissue deformation with helical growth and left-handed deviation of the root (Fig 2B). Moreover, during the first stages of growth, farnesene-treated *A*. *thaliana* seedlings showed an extreme apparent loss of the gravitropic response (agravitropism) with horizontal growth, curling, and occasionally upward growth of roots (Fig 2C).

### Assay for root gravitropism

Since the experiment on the morphology of farnesene-treated roots pointed out a strong alteration of the normal gravitropic response and an alteration of normal root anatomy, an assay to evaluate the response of gravistimulated root was carried out in order to highlight if the alteration observed was due to a loss of gravitropic perception or to the alteration of cell anatomy.

**Fig 3:**
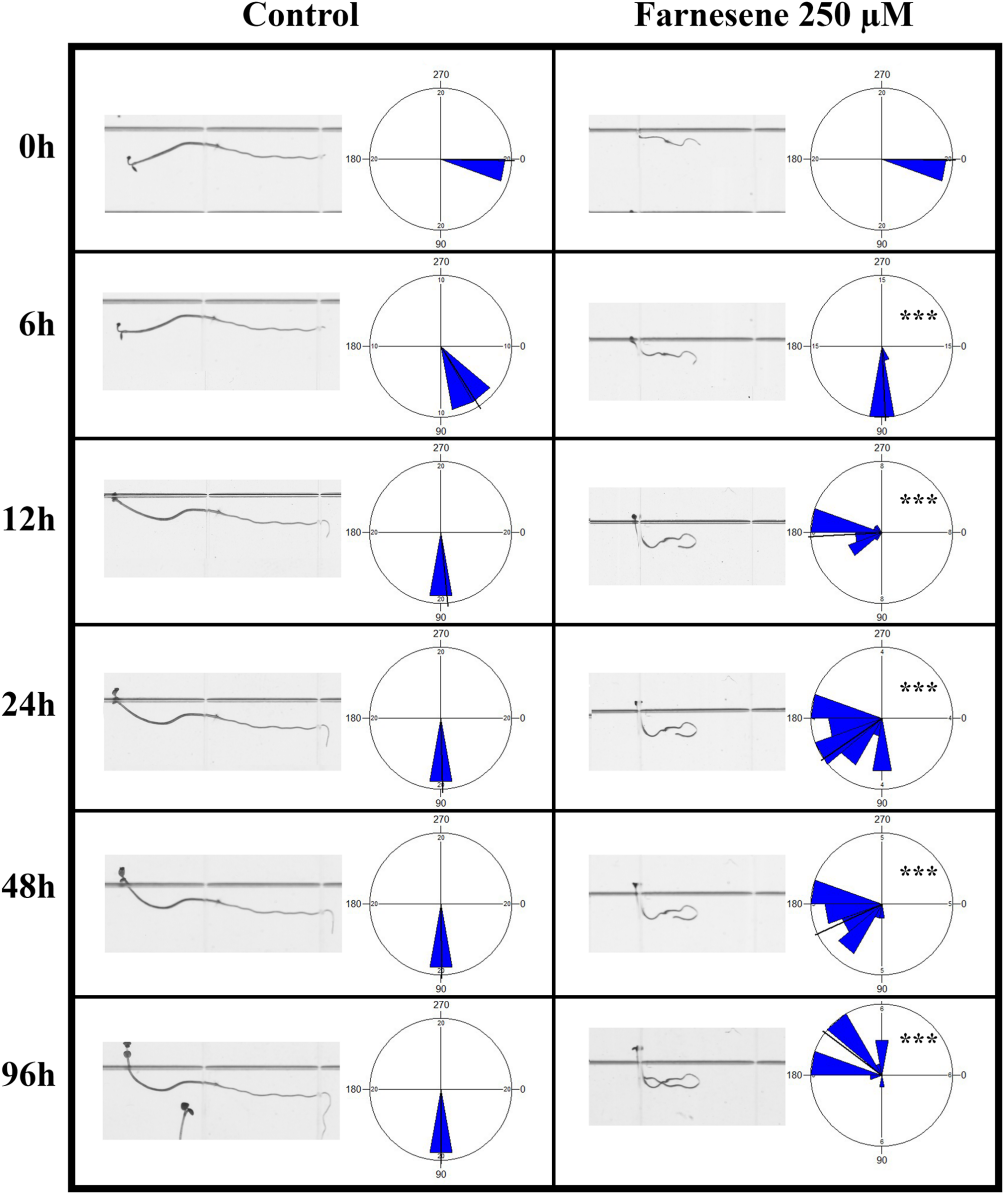
Bioassay on root gravitropism. Effect of farnesene on the time course gravitropic curvature of *Arabidopsis thaliana* primary roots. After 90° rotation, images were taken after 6, 12, 24, 48 and 96 hours. Data were analyzed through t-test (*P* ≤ 0.05). Circular graphs indicate the distribution of the angle of root curvature. * = (*P* ≤ 0.05), ** = (*P* ≤ 0.01), *** = (*P* ≤ 0.001). N = 10.

The experiments carried on gravistimulated seedlings pointed out that farnesene extremely altered the normal tropism of *A*. *thaliana* roots. In particular, the gravitropic bending of the root apex in control seedlings was already visible after 6 hours of treatment and reached ≈ 90° of bending after 12 hours maintaining this curvature for the rest of the experiment. By contrary, although farnesene-treated seedlings were able to perceive in a relatively short period (6 h) the gravitropic stimulus, they gradually lost it (Fig 3). Interestingly, along the 96 h of observation, farnesene seedlings were successively perceiving gravity and loosing the stimulus, which resulted in an abnormal and random pattern of growth (Fig 3).

### Taxol reversal bioassays

Furutani [20] reported that the addition of propyzamide (a microtubule destabilize left-hand inducer) to agar medium was able to restore the normal growth direction of right hand mutants (*spr1*), confirming the pivotal role played by microtubules on the anisotropic growth of roots. Therefore we have tried to invert the left-handed growth caused by farnesene using taxol, a chemical known to induce right-hand growth on *A*. *thaliana* roots.

**Fig 4:**
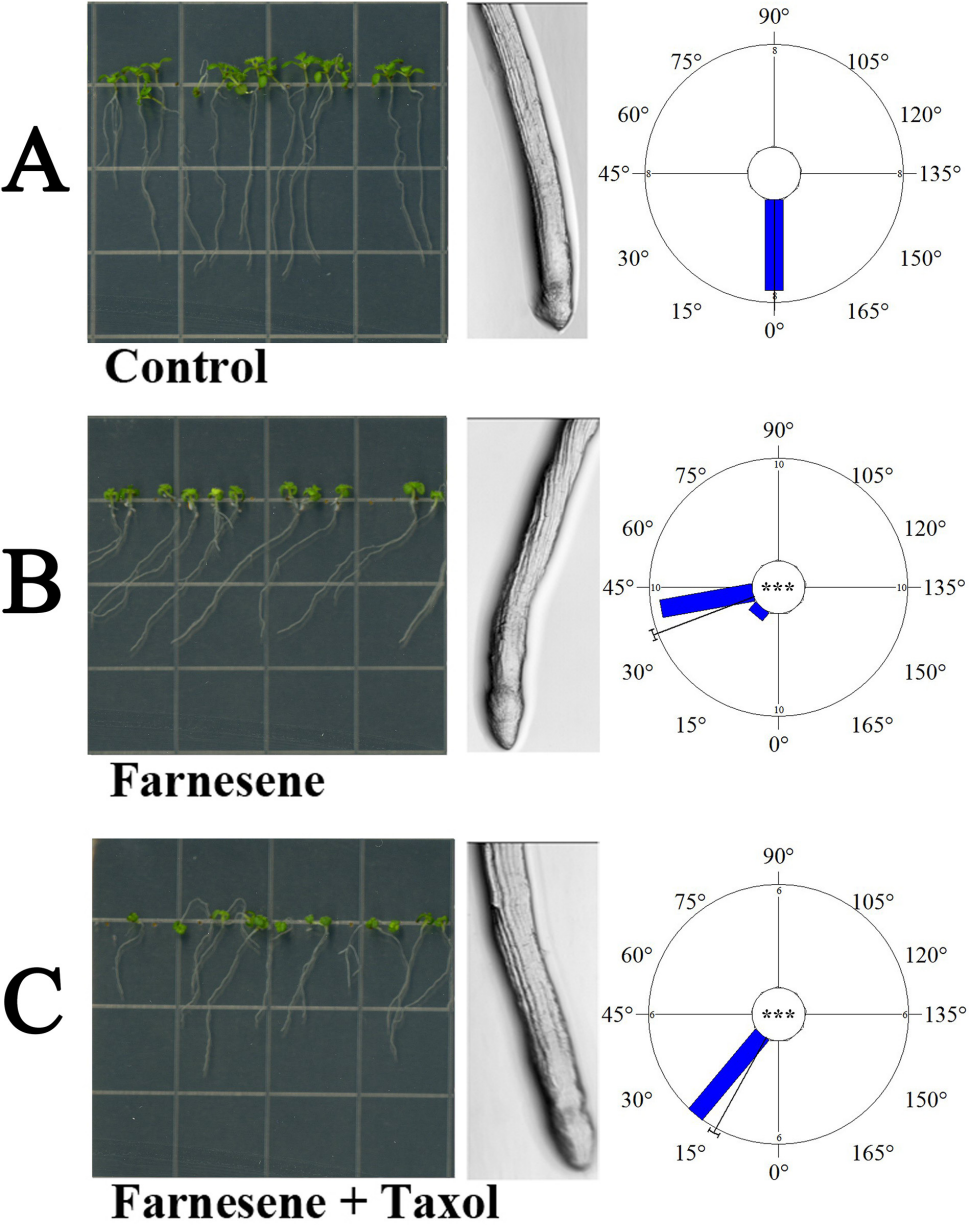
*Arabidopsis* seedlings grown in taxol and/or farnesene for 14 days. A) Seedlings at time zero, when treatment started; B) Untreated (control); C) 250 μM farnesene; D) 250 μM farnesene + 0.5 μM taxol. Circular graphs indicate the angle of root curvature. * = (*P* ≤ 0.05), ** = (*P* ≤ 0.01), *** = (*P* ≤ 0.001). Images magnification 20X. N = 5.

As shown in the circular graphs of Fig 4, *A*. *thaliana* roots treated with 250 μM farnesene showed a left-handed deviation of 34° when compared to the normal gravitropic response of the control. The addition of taxol, a well-known microtubule stabilizer, significantly reversed this effect restoring the gravitropic response and reducing left-handed deviation (14°) (Fig 4).

### Morphological analysis of the root cell structure

The macroscopic effects observed on plant roots after a treatment are an expression of microscopic alterations at cellular level. The study of cells by TEM can give a hint regarding what is happening in the plant and how it is reacting to stress factors.

Ultrastructure TEM analyses of untreated and farnesene-treated *A*. *thaliana* roots showed that farnesene strongly alters the root cell structure and organization. A summary of the overall effects is reported in Table 1, whereas the quantification of some of the altered parameters observed in farnesene-treated roots is reported in Table 2.

**Table 1:** Effects of 250 μM farnesene on the ultra-structure of 7 and 14 days treated roots. Cell ultra-structure and changes in the organelles were identified analyzing transmission electron microscopic images of farnesene-treated and untreated roots. A number of 200 images were analyzed for this table.

	250 μM farnesene	
	7 days	14 days
Cell division	Irregular nuclei	
	Irregular and incomplete cell division	
	Multinucleated cells	
		Abnormal cell shape
		Incomplete phragmoplast
Cytoplasm	Cytoplasm degradation	
Cell wall	Consistent effects on cell wall: Irregular cell wall and plasmodesmata	
	Aberrant cell walls	
	Cell wall deposits and electro-dense deposits	
	Plasmatic membrane detached from the cell wall	
Golgi complexes	Vesicles and deposits: Increased Golgi number	
		High number of vesicles
Microtubules	Reduction of the microtubules number	
Mitochondria	Broken mitochondria	
		Mitochondria with swollen/translucent stroma
		Cluster of mitochondria
Vacuoles	Increase in size and number of the vacuoles	
	High vacuolation	
	Endocytosis	

**Table 2:** Quantification of changes detected in the cell structure and the organelles of 250 μM farnesene-treated and untreated (0 μM) *Arabidopsis* roots. A number of 100 TEM images was used for the quantification of each parameter.

Observations	7 days treatment		14 days treatment	
	0 μM	250 μM	0 μM	250 μM
**Cell wall thickness**	0.116 ± 0.018	0.26 ± 0.047***	0.121 ± 0.019	0.33 ± 0.093**
**Density of plasmodesmata**	0.61 ± 0.13	0.22 ± 0.08***	0.59 ± 0.11	0.19 ± 0.07***
**Density of polynucleated cells**	0 ± 0	0.032 ± 0.009***	0 ± 0	0.04 ± 0.026***
**Density of abnormal nuclei**	0 ± 0	0.071 ± 0.008***	0 ± 0	0.064 ± 0.018***
**Density of cell wall deposits**	0.028 ± 0.005	0.259 ± 0.015***	0.087 ± 0.07	0.334 ± 0.041***
**Vacuole size**	4.004 ± 1.407	9.924 ± 2.80**	4.035 ± 1.21	9.732 ± 1.56***
**Density of mitochondria**	0.275 ± 0.05	0.492 ± 0.051***	0.276 ± 0.035	0.446 ± 0.107*
**Density of broken mitochondria**	0 ± 0	0.03 ± 0.01***	0 ± 0	0.053 ± 0.015***
**Density of microtubules**	24.31 ± 5.588	1.275 ± 0.916**	22.11 ± 0.795	0.795 ± 0.357***
**Microtubules distance from cell wall**	0.021 ± 0.007	0.028 ± 0.002	0.02 ± 0.008	0.03 ± 0.01

Ultrastructure measurements were expressed as: cell wall thickness (μm); Density of plasmodesmata (number of plasmodesmata / μm of cell wall); Density of polynucleated cells (number of polynucleated cells / 100 cells); Density of abnormal nuclei (number of aberrant nuclei / 100 cells); Density of cell wall deposits (number of cell wall deposits / 100 cells); Vacuole size (μm^2^); Density of mitochondria (number of mitochondria / cell); Density of broken mitochondria (number of broken mitochondria / cell); Density of microtubules (number of microtubules / μm of cell wall); Microtubule distance from cell wall (μm). Data were expressed as mean ± SD. Differences between treatments were statistically analyzed through t-test with *P* ≤ 0.05.

* = (*P* ≤ 0.05),

** = (*P* ≤ 0.01),

*** = (*P* ≤ 0.001).

**Fig 5:**
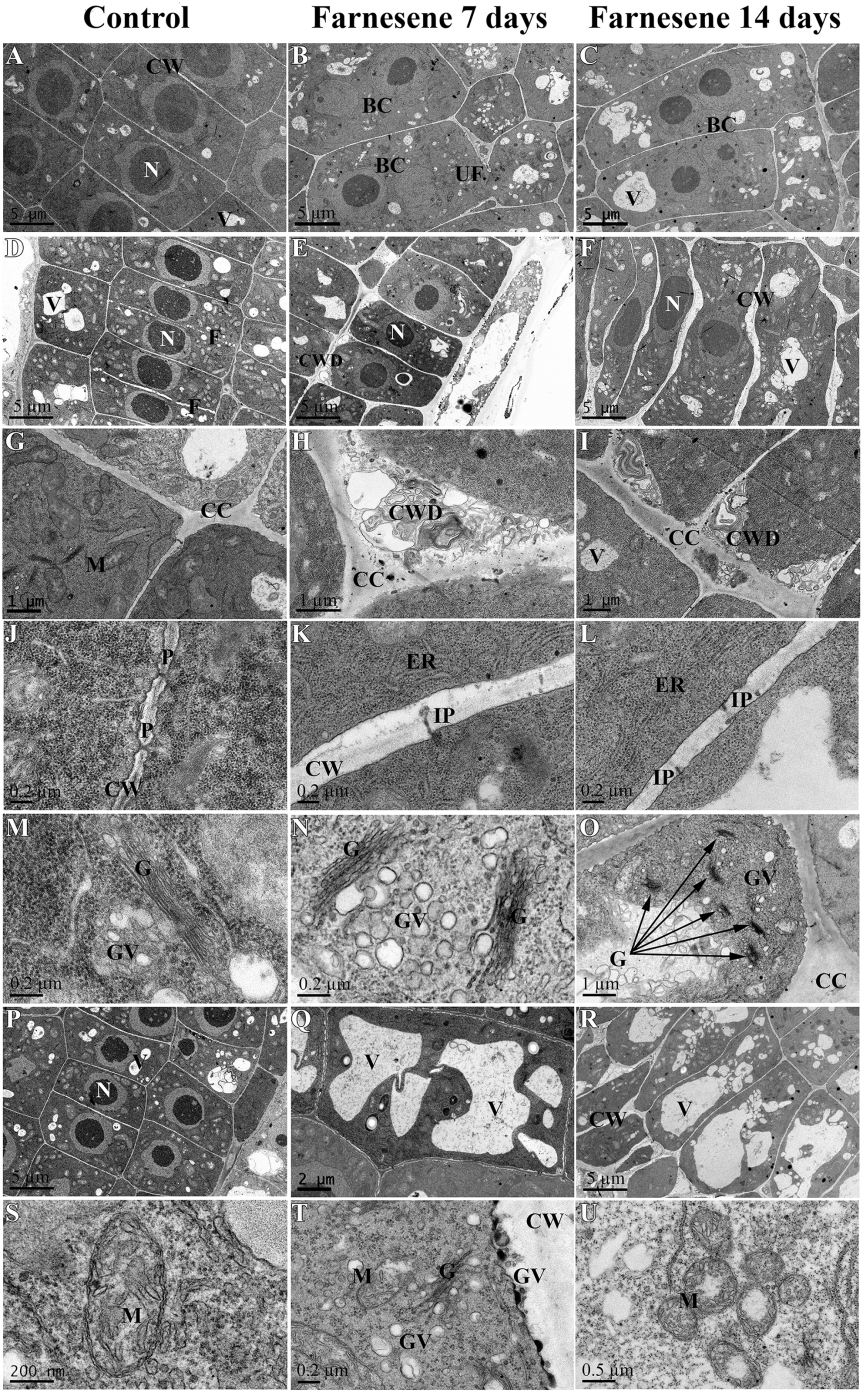
TEM images of farnesene-treated and untreated *Arabidopsis* meristems. TEM images of the apical meristem of untreated (A, D, G, J, M, P, S), and 7 days (B, E, H, K, N, Q, T) and 14 days (C, F, I, L, O, R, U) farnesene-treated *Arabidopsis* roots: A) Nucleus of a stele cell; B) Binucleated cell of a stele cell; C) High presence of polynucleated cells; D) Epidermal cells with normal cell division and phragmoplast formation; E) Epidermal cells with high presence of cell wall deposits on cell corners; F) Protodermal cells with abnormal shape and swollen cell walls; G) Normal cell corner without deposits; H and I) High presence of cell wall deposits; J) Plasmodesmata; K and L) High presence of incomplete plasmodesmata; M) Regular Golgi apparatus; N) Golgi apparatus with massive production of vesicles; O) High presence of Golgi apparatus and vesicles; P) Normally vacuolated cortex and endodermic cells; Q and R) Endodermal cell: increase in size and number of the vacuoles; S) Mitochondria with regular morphology and a good number of cristae; T) Broken mitochondria, High number of Golgi vesicles; U) Increased number of mitochondria characterized by irregular shape and swollen/translucent stroma. Nucleus (N), vacuole (V), cell wall (CW), mitochondria (M), binucleated cell (BC), phragmoplast (F), cell wall deposit (CWD), cell corner (CC), plasmodesmata (P), endoplasmic reticulum (ER), incomplete plasmodesmata (IP), Golgi apparatus (G), Golgi vesicles (GV), uncompleted phragmoplast (UF).

TEM analyses of control cells revealed that elongation and differentiation zones were characterized by symmetric rows of cells with regular cell anatomy (Fig 5A, 5D, 5G, 5J, 5M, 5P and 5S). Root cells were aligned and maintained a symmetric pattern of division. Moreover, the meristematic cells of control roots did not show any aberration in cell organization after either 7 or 14 days of growth (Fig 5A, 5D, 5G, 5J, 5M, 5P and 5S).

On the contrary, TEM images of treated roots showed highly disorganized meristems with asymmetric tissue patterns (S1 Fig) and alteration of cell structure, highlighting the strong phytotoxic effect exerted by farnesene (Fig 5B, 5C, 5E, 5F, 5H, 5I, 5K, 5L, 5N, 5O, 5Q, 5R, 5T and 5U).

As reported in Fig 5B and 5C and Table 2, root cells treated with farnesene for 7 and 14 days were characterized by a high presence of binucleated cells and aberrant phragmoplasts. Farnesene treatment (7 and 14 days) caused consistent effects on cell wall (Fig 5F, 5I, 5K, 5L and 5R). In fact, treated plants were characterized by cell walls that were 2.3 (7 days) and 2.8 (14 days) times thicker than in control cells (Table 2). Cell wall alteration was accompanied by a strong alteration on plasmodesmata structure (Fig 5K and 5L) and density, which was ≈ 63% and 67% lower than control after both 7 and 14 days of treatment, respectively (Table 2). Moreover, a high presence of cell wall deposits was observed in the cell corners of treated cells (Fig 5H and 5I), which were 9.5 (7 days) and 5 (14 days) times more frequent than in control roots (Table 2).

Another characteristic of farnesene-treated cells was the increment of vacuole number and/or size (Fig 5Q and 5R), which was 2.5 times higher than the control (Table 2).

Moreover, on both 7 and 14 days treated cells were observed broken (Fig 5T) and condensed mitochondria with swollen/translucent stroma (Fig 5U), which were increasing in number (80 and 60% respectively, compared to the control; Table 2) and clustering (Fig 5U).

As well, cells with high presence of Golgi apparatus and vesicles production (Fig 5N and 5O) were also observed after both 7 and 14 days of treatment.

Finally, as reported in Table 1 and S2 Fig, tetraploid cells, electro-dense cell wall deposits, high presence of vesicles in the cytoplasm and on cell wall, autolytic activity and clear signs of cytoplasmic degradation were also observed.

### Microtubules visualization

Since farnesene treatments caused an anisotropic growth of the root, which suggests an alteration of the microtubule organization, TEM and immunolocalization analyses were carried out on untreated and treated roots in order to visualize their presence and distribution and to understand their involvement in the mode of action of farnesene.

**Fig 6:**
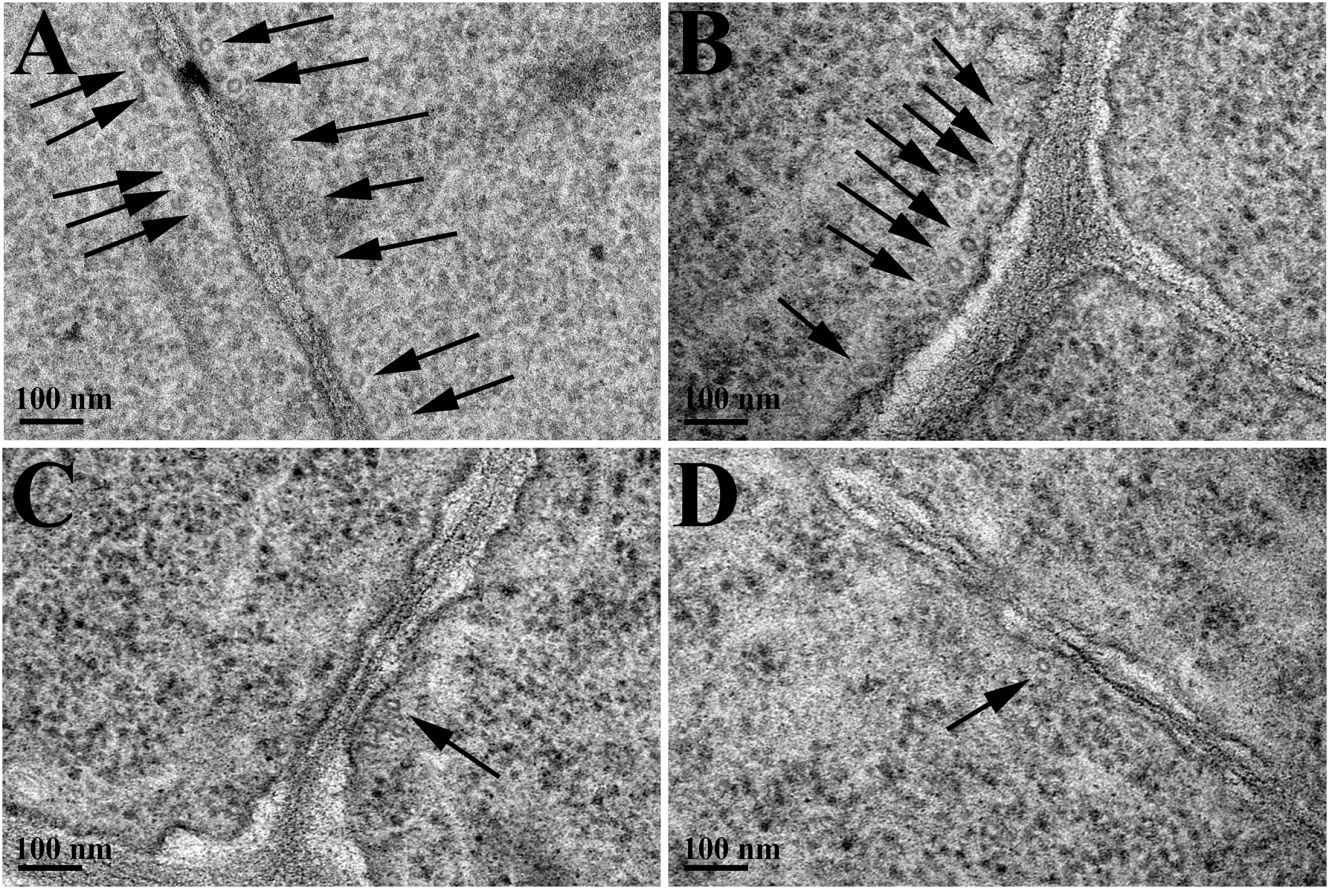
TEM images of cortical microtubules. TEM images of cortical microtubules (transversal section) in cells of *Arabidopsis* roots grown in 250 μM farnesene for 7 and 14 days. A) Cell of 7 days control roots; B) Cell of 14 days control roots; C) Cell of 7 days farnesene-treated roots; D) Cell of 14 days farnesene-treated roots. Black arrows indicate transversally cut microtubules.

Microtubules were found in both transverse and longitudinal sections after TEM of control roots (Fig 6). In transverse sections, microtubules were mainly observed in parallel groups arranged to the cell wall, morphologically homogeneous and always maintaining the same distance to the cell wall (Fig 6A and 6B).

By contrary, in transverse sections of farnesene-treated roots, microtubules were only observed in small groups, frequently isolated or in pairs, and were less visible than in control roots in the analyzed TEM preparations (Fig 6C and 6D). Interestingly, the distance from microtubules to the cell wall was unaltered (after 7 and 14 days) either in the control or in the treatment, whereas microtubule density was extremely reduced in treated roots (Table 2). In fact, in 7 and 14 days treated roots, microtubule density was 94 and 96% lower than the control, respectively (Table 2).

**Fig 7:**
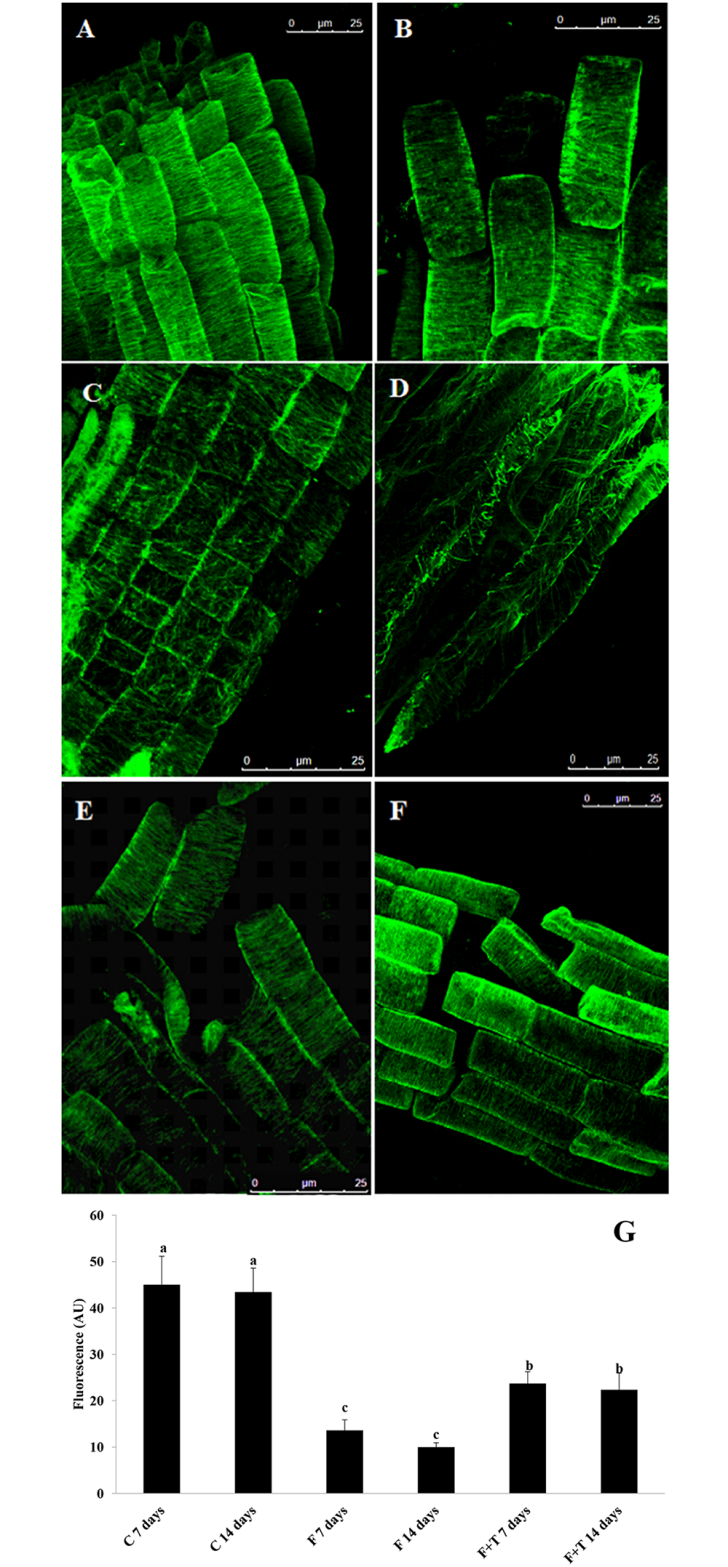
Microtubule immunostaining of farnesene and taxol treated and untreated *Arabidopsis* roots. Figure shows (A) control cells after 7; and (B)14 days; (C) cells treated with 250 μM farnesene for 7 and (D)14 days; (E) cells treated with 250 μM farnesene + 0.5 μM taxol for 7 and (F)14 days. Images were acquired by confocal microscopy (63X immersion objective). (G) Fluorescence quantification of immunolabeled microtubules of roots treated for 7 and 14 days: control (C); farnesene 250 μM (F); taxol 0.5 μM (T). Data are expressed as arbitrary units (AU). Different letters between the bars indicate significant differences at *P* ≤ 0.05 (LSD test). N = 5. Fluorescence was quantified using the open source software ImageJ.

After immunolabeling of control roots, microtubules were observed well-defined and typically arranged, being parallel to the transverse axis of the cells, and uniform in density at both 7 and 14 days of experiment (Fig 7A and 7B). By contrary, immunolabeling of farnesene-treated roots showed erratically arranged microtubules after 7 and 14 days of treatment (Fig 7C and 7D), with evident loss of symmetry and reduced in density (Fig 7G), which was even more evident after 14 days of treatment with values of fluorescence intensity reduced in almost a 70% in farnesene-treated roots when compared to the control (Fig 7D and 7G). Interestingly, the combined treatment of farnesene and taxol was able to restore the control-like arrangement of the microtubules after 7 and 14 days. Although microtubules density was still affected after both times of treatment, values were higher than in farnesene-treated roots (Fig 7E, 7F and 7G).

### Effects on auxin and ethylene content

The alterations induced by farnesene on root morphology (bold root, lateral root inhibition, left-handedness, etc.) suggest an alteration of the cross-talk between auxin and ethylene, whose implication in root morphology regulation is largely known. Therefore, we decided to quantify their presence in untreated and treated roots in order to understand their role in farnesene’s effects.

**Fig 8:**
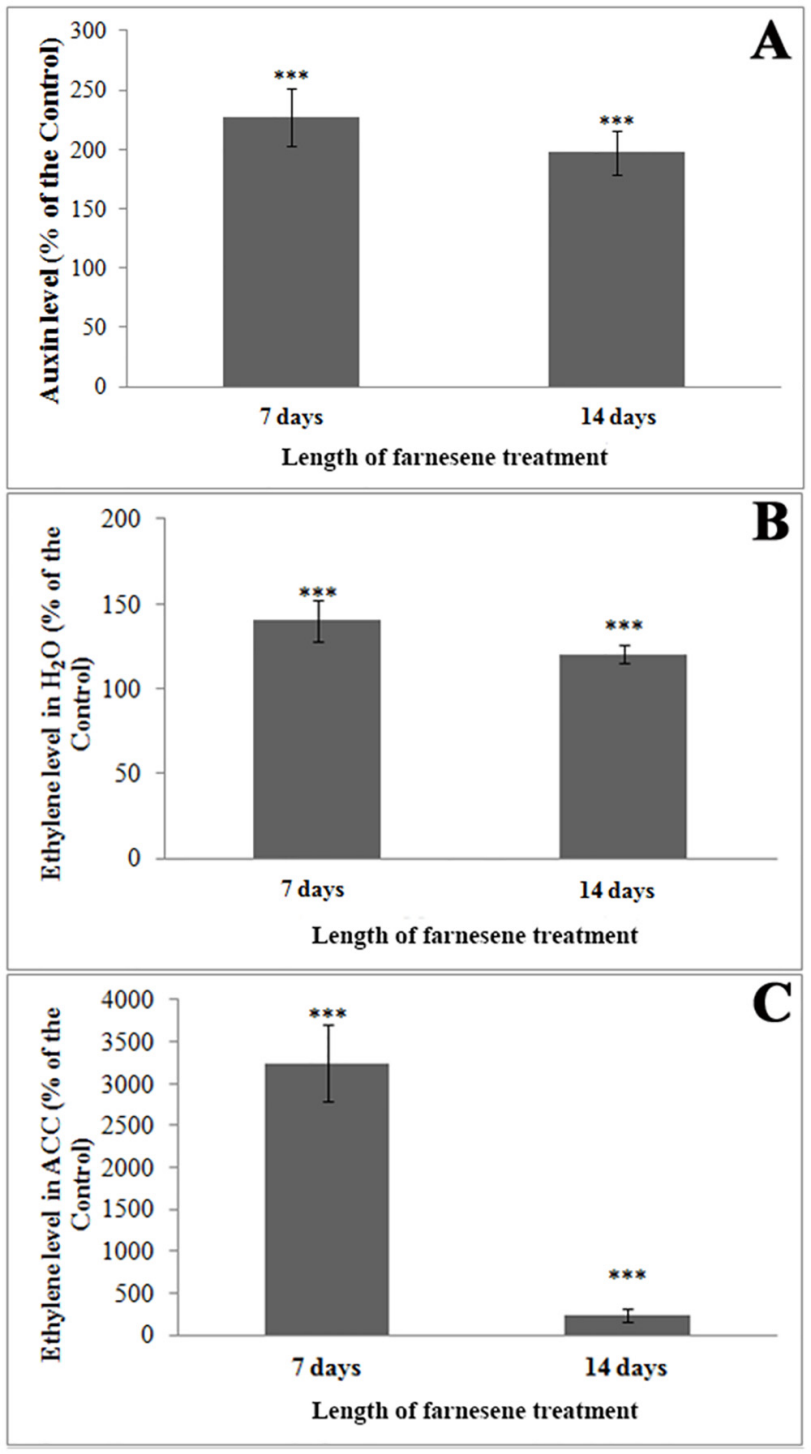
Auxin and ethylene contents of farnesene-treated and untreated seedlings. A) Auxin concentration; B) Ethylene emission after incubation in distilled water (real level); C) Ethylene emission after incubation in ACC (ACC addition leads to obtain maximal ethylene emission) in *A*. *thaliana* roots after 7 and 14 days treated with 250 μM farnesene. Data are expressed as percentage of the control. * = (*P* ≤ 0.05), ** = (*P* ≤ 0.01), *** = (*P* ≤ 0.001). N = 6.

Interestingly, auxin content significantly increased in *A*. *thaliana* roots treated with 250 μM farnesene reaching values 1.2 and 0.9 times higher than control roots after 7 and 14 days treatment, respectively (Fig 8A).

Ethylene content showed also a similar trend to auxin (Fig 8), with increases of 0.4 and 0.2 fold compared to the control after 7 and 14 days of treatment, respectively (Fig 8B and 8C). After the incubation with the ethylene precursor aminocyclopropane carboxylic acid (ACC), there was a significant increase of ethylene production 7 days after treatment (32 fold higher than control) (Fig 8C). Although the ethylene level strongly decreased after 14 days treatment, it was still significantly higher than the control.

### In situ semi-quantitative determination of H_2_O_2_, O_2_^−^ and NO emission

As well as hormone balance also ROS and RNS production could be induced and regulated by phytotoxins. Moreover, it’s largely known that the accumulation of ROS can affect both plant hormonal equilibrium [31, 33, 34] and root morphology [32] as well as cell cycle progression and microtubule organization [42]. It is known that cellular extension is partially under the control of microtubule network. Oxidative stressors and auxin [43, 44] can alter microtubules orientation through the activation of specific MAP-kinases. As a consequence, microtubule organization and cell elongation dynamics could be altered creating a link between oxidative stress and plant architecture.

**Fig 9:**
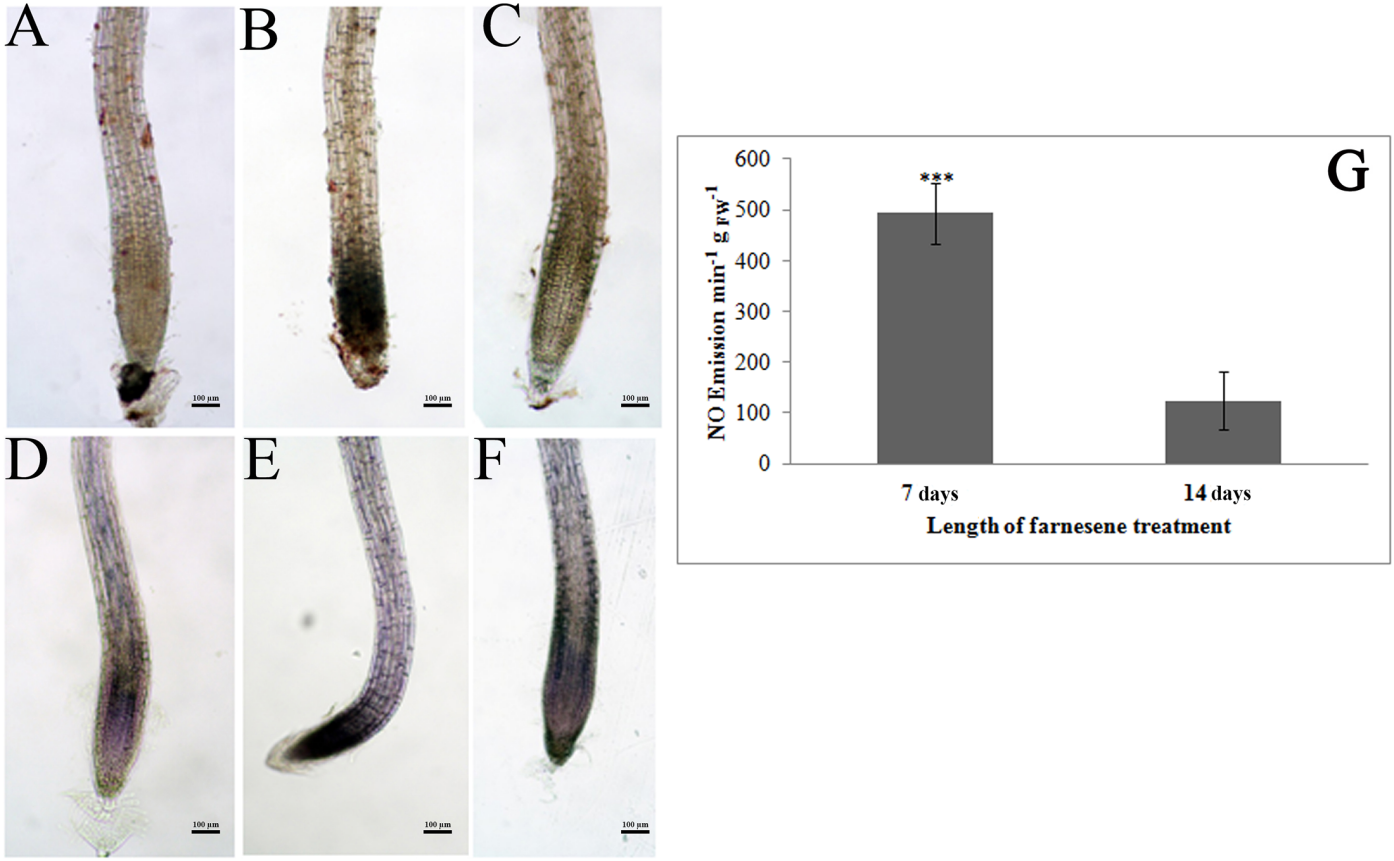
*In situ* hydrogen peroxide and superoxide localization in farnesene-treated and untreated roots. *In situ* hydrogen peroxide (A-C) and superoxide (D-F) localization in roots of *Arabidopsis* treated with 250 μM farnesene for 7 and 14 days. Control (a and d), 7 days (b and e), 14 days (c and f). (G) NO emission (expressed as arbitrary units) from *Arabidopsis* roots treated with 250 μM farnesene for 7 and 14 days. * = (*P* ≤ 0.05), ** = (*P* ≤ 0.01), *** = (*P* ≤ 0.001). Image magnification 12X. N = 3.

*A*. *thaliana* seedlings treated with 250 μM farnesene showed a marked production of H_2_O_2_ and O_2_^-^, especially after 7 days (Fig 9B and 9E). This increase of ROS is indicative of altered cellular redox equilibrium and potential oxidative stress. However, there was a substantial recovery of this equilibrium after 14 days treatment, with values of H_2_O_2_ and O_2_^-^ similar to those of control roots (Fig 9C and 9F).

The NO emission, measured as increase in DAF fluorescence, was also detected in *A*. *thaliana* roots treated with 250 μM farnesene for 7 and 14 days (Fig 9G). While NO emission was relatively low in control roots, there was a significant increase of DAF fluorescence (3.93 fold higher than control) after 7 days farnesene treatment. Again, no differences in NO emission were detected between control and treated roots after 14 days (Fig 9G).

## Discussion

The effects here reported of farnesene on *A*. *thaliana* seedlings included strong inhibition of root growth, modifications of root morphology, important tissue alterations and cellular damages. Primary root growth, lateral root number, lateral root length and both root hairs length and density were remarkably reduced, while cotyledons and seedling rosette leaves were weakly affected (at least up to 14 days of evaluation). These inhibitory effects on root growth have been widely reported in plants exposed to allelochemicals [15, 45], including sesquiterpenes such as dehydrozaluzanin C, that strongly affect plasma membrane [46]. In the present work, the potential mode of action of farnesene on root system was proposed.

Our results showed that farnesene induced a reduction in lateral root formation together with an increment of endogenous auxin and ethylene levels in treated *A*. *thaliana* roots, especially after 7 days of treatment.

It is well known that ethylene and auxin, agonistically or antagonistically, regulate root morphology in *A*. *thaliana* plants [47–50]. Furthermore, it is known that ethylene stimulates acropetal auxin transport in *Arabidopsis* roots, which is linked to a reduction of lateral root formation in a PIN3- and PIN7- (efflux auxin carriers) dependent way [51]. Recent studies underlined that some allelochemicals such as coumarin and 4-methylumbelliferone (4-MU) could have an auxin-like behavior or/and interact with the auxin signaling or redistribution [52, 53]. Ivanchenko et al. [54] confirmed the synergistic role played by auxin and ethylene in inhibiting lateral root initiation. Moreover, they demonstrated the ability of ethylene to promote the emergence of existing lateral root primordials, while at the same time high concentrations of endogenous ethylene inhibit the ability of pericycle cells to initiate new lateral root primordia. These results are in accordance with the increment of lateral roots observed in farnesene-treated seedlings in previous experiments [55], where *A*. *thaliana* seedlings were treated with farnesene when they were 7 days-old and, consequently, they had already initiated the development of lateral roots.

As well, a marked increment in ethylene production was also observed in the presence of ACC (the immediate precursor of ethylene) after 7-d farnesene treatment, which could be due to higher activity of the ACC oxidase, known to be increased during stress conditions [56, 57]. This effect was accompanied by increases of ROS and NO levels in the treated roots. These data are supported by the observation of Gniazdowska et al. [58], which reported that the addition of H_2_O_2_, NO or hydrogen cyanide (HCN) into ACC water solution in the absence of any plant material resulted in a large quantity of ethylene formation. After 14 days of treatment, the reduction of ethylene production in the presence of ACC (although statistically higher than the control) was followed by a reduction of NO emission and ROS production. These results also confirmed the participation of these bioactive molecules in lateral root development and their involvement in cross-talk with phytohormones, as already reported by Correa-Aragunde et al. [59] and Yu et al. [32]. Previous experiments demonstrated that root meristem size is altered not only by individual ROS concentrations but also by H_2_O_2_/O_2_^-^ ratio [60]. In this way, Dunand et al. [61] found that the distribution of superoxide and hydrogen peroxide in *A*. *thaliana* roots strongly influenced root development and, especially, root hair formation and growth. Over-accumulation of H_2_O_2_ (particularly observed under stress conditions) led to modification of root shape (reduction of root length and root meristem) and decreased root hair number in *A*. *thaliana* [60, 62]. Increased H_2_O_2_ and O_2_^-^. levels in root tips were observed after 7 days of *A*. *thaliana* treatment with farnesene. Similar ROS accumulation was also detected in *A*. *thaliana* roots treated with p-hydroxybenzoic acid and in onion (*Allium cepa* L.) or maize (*Zea mays* L.) roots grown in the presence of cyanamide [63–65]. Moreover, in accordance to our results, Romero-Puertas et al. [66] observed that the auxinic herbicide 2,4-dichlorophenoxyacetic acid (IAA analogue) caused root growth inhibition accompanied by H_2_O_2_ and O_2_^-^ overproduction.

These results indicate that the mode of action of farnesene is related to the alteration of the hormonal balance and the consequent stimulation of ROS accumulation in acceptor plants. Burst of NO in plant tissues, as that observed in farnesene-treated roots, is also a common response to different stressors [32]. However, involvement of NO in the response of acceptor plants to allelochemical compounds is rather poorly known. Recently, rapid induction of NO emission after treatment of *A*. *thaliana* seedlings with p-hydroxybenzoic acid was also demonstrated [63]. These results pointed on NO participation as a common indicator of a response reaction of *A*. *thaliana* roots to phytotoxins. Alteration in NO concentration affects actin cytoskeleton, as it has been demonstrated for maize roots apices [67]. Formation of endocytic vesicles and trafficking in cortex cells of the transition zone of maize root apex were affected by NO application. Moreover, it has been shown that the level of PIN1 proteins, which participate in acropetal auxin transport, was reduced by NO [68].

Auxin accumulation could also mediate F-actin disruption, as observed with 4-MU [52] on the primary root of *A*. *thaliana*, resulting in malformation of the apical meristem. Moreover, as observed by Walne et al. [69], high auxin levels strongly damage the cell ultra-structure resulting in cytoplasmic vacuolation, dictyosomes with swollen cisternae and strong production of vesicles. According to these results, the accumulation of endogenous auxin induced by farnesene in root resulted in strong ROS production, alteration of the cell ultrastructure and microtubules disorganization, accompanied by root malformation, anisotropic root growth and lack of root hair production. These results are in agreement with the one obtained for tomato (*Solanum lycopersicum*) roots treated with cyanamide. Phytotoxic effects of this compound were related to the modifications in cell division and increased IAA levels [70].

Interestingly, the macroscopic effects observed on farnesene roots could be due to the ultra-structural damages potentially caused by auxin accumulation. In fact, roots treated with farnesene assumed a corkscrew shape characterized by a torsion with a consistent direction called "handedness" and a loss in gravitropic response, already observed with this molecule in previous studies [15]. In particular, farnesene induced left-handedness growth of *A*. *thaliana* roots, suggesting that this sesquiterpene could be acting on microtubules, as previously observed for the microtubule stabilizer taxol (right handedness) or the microtubule disruptor propizamyde (both depolymerizing drugs) [16]. It has been demonstrated that mutants showing left-handedness exhibited defects on microtubules arrangement, in particular negative mutations at the tubulin intradimer interface of α-tubulins 4 and 6 [19]. On the contrary, mutants whose roots showed wavy random movements or coil formation, seemed to have deficiencies in auxin homeostasis [18]. These results suggest that the ultrastructure alteration, probably due to auxin accumulation, and the macroscopic effects observed on root structure as left-handedness are connected. In fact, the effects observed during the gravistimulation bioassay pointed out that treated seedlings were still able to perceive the gravitropic stimulus, although the alteration of the cellular shape, mainly present in the elongation zone, was the main factor that was mediating the loss of the gravitropic response. This effect could be due to both auxin and/or ethylene accumulation. In fact, it is known that endogenous ethylene accumulation negatively influences gravitropic movements of roots [71, 72].

Moreover, farnesene, through the stimulation of endogenous auxin production, could interfere with microtubule dynamics by acting as a stabilizer of the tubulin monomers or polymers, modifying the proper orientation of microtubules.

Microtubules are essential components of cell cytoskeleton and they are involved in crucial processes, including division, growth and differentiation. These activities depend on a highly sensitive and dynamic balance between the different subunits that form the microtubules [73]. When this balance is altered, the orientation of microtubules in the cell is affected, as well as all the cellular processes depending on them. In this work, clear evidences of altered microtubule organization after farnesene treatment were observed.

The irregular distance between microtubules and the small groups that they form in the proximity of the cell wall may indicate a disruption of cortical microtubules. When the typical disposition of microtubules, which are perpendicularly aligned to the primary cell wall, is disrupted, cellulose microfibrils arrangement is disorganized, cells do not elongate properly [74, 75], and consequently there is an altered cell differentiation [76].

Besides, the presence of multinucleated cells in farnesene-treated seedlings suggests an improper formation of phragmoplasts, resulting in aberrant cell divisions. Similar effects were found after treatment with other well-known mitotic disrupter drugs such as griseofulvin and the carbamate herbicides IPC or CIPC. Plant cells treated with these chemicals showed abundant spindle microtubules at prometaphase, oriented toward many poles, resulting in multinucleated cells with walls formed in multiple planes, instead of a single plane of division [77]. Other compounds, like caffeine, theophylline, caffeic acid, induce also abnormal phragmoplast formation [77] with a morphology similar to the farnesene-treated cells, where vesicles could not arrange correctly in the cell plate to form the new cell wall.

Moreover, the evaluation of the spatial arrangement of cortical microtubules in farnesene-treated plants confirmed that this sesquiterpene is able to alter cortical microtubules organization in a way similar to that observed in plants treated with other chemicals [20, 78]. Interestingly, as reported by Furutani et al. [20] on spiral mutants (*spr1* and *spr2*), the exogenous application of drugs, such as taxol or propyzamide, reverted the direction of the epidermal helix in a dose-dependent manner suppressing the cell elongation defects of spiral seedlings. Similar response was observed in farnesene-treated *A*. *thaliana* roots after exogenous addition of taxol, which significantly restored microtubule arrangement, root cell alignment and, consequently, normal root growth direction. Thus, these results, joined to the TEM observations on microtubules organization and their spatial disposition, support the initial hypothesis that the altered direction of root growth and the malformations in roots treated with farnesene are due to its activity on cortical microtubules, which affect root cell isotropy as also described by Hashimoto [16]. It was observed that chemicals interacting with tubulin, such as oryzalin or colchicines, generally cause two different effects on *A*. *thaliana* roots, radial expansion and root growth inhibition [79]. On the other hand, farnesene pointed out a strong root growth inhibition without any stimulation of radial expansion, which was only observed after exposure to higher doses. Probably, the highest concentration of farnesene affected both mitotic [24] and cortical microtubules. Moreover, it is largely known that cortical microtubules are among the most resistant to chemical effectors, whereas spindle and phragmoplast microtubules are the most sensitive [80]. These results, strongly supported by bibliography, suggest that farnesene could act as a mitotic disrupter bioherbicide inhibiting the formation of both cortical and mitotic microtubules [24]. Based on the classification of mitotic disrupter herbicides, farnesene could be included in the third group, as the phosphoric amides. These compounds affect the formation and organization of all microtubules resulting in the typical swollen cellular morphology, abnormal cell wall formation and large irregular nuclei after abortive arrest of mitosis also observed in farnesene-treated cells and principally due to the effects on cortical microtubules, disruption of mitotic microtubule organizing centers and the absence of mitotic microtubule arrays. Moreover, farnesene-treated cells showed aberrant phragmoplasts, binucleated and tetranucleated cells and misplaced cell walls, typically found in roots treated with microtubule inhibitors [24, 80].

Taken together, these results show that farnesene alters the hormonal balance, inducing an increment of both auxin and ethylene content, and stimulates oxidative damage through the production of NO, H_2_O_2_ and O_2_^-^. Both hormones and ROS could alter the normal root growth interfering with cell division and cytokinesis through disruption of mitotic and cortical microtubules and by inducing ultra-structural cell malformations and consequent alterations on root morphology through altering the cross-talk between auxin and ethylene. The discovery of natural molecules, such as farnesene, characterized by strong phytotoxic potential is pivotal for the development of bioherbicides able to overcome the resistance phenomenon in weeds.

In depth studies on the dynamics and stability of this compound into the plant will be welcome in the future to fully understand its effects on plant metabolism and improve its use for weed management.

## Supporting Information

S1 Fig TEM panoramic view of farnesene treated *Arabidopsis* meristem. A) altered meristem of farnesene treated plant; B) Particular view of the meristem reported in figure A (black square). Note the altered organization of the meristem as well as the high presence of cells with uncompleted division. Nucleus (N), vacuole (V), uncompleted phragmoplast (UF). Similar images were observed after both 7 and 14 days of treatment.
(JPG)

S2 Fig TEM microphotographs of farnesene-treated *Arabidopsis* meristems. TEM microphotographs of the apical meristem of 7 days (A, C, E) and 14 days (B, D, F) treated *Arabidopsis* roots.: A) Signs of active exocytosis and mitochondria with irregular shape and translucent stroma; B) Tetranucleated cell showing incomplete cell wall; C) vacuoles autophagy activity; D) Electro-dense deposit on cell wall); E) Degradation of the cytoplasm in the elongation zone and plasmatic membrane detached from the cell wall; F) Protodermal cells with incomplete phragmoplast, abnormal shape and swollen cell walls; G) and H) Degraded cytoplasm and mitochondria with irregular morphology and translucent stroma. Nucleus (N), vacuole (V), cell wall (CW), mitochondria (M), incomplete plasmodesmata (IP), Golgi vesicles (GV), uncompleted phragmoplast (UF), tetranucleated cell (TC), electro-dense deposits (ED), amyloplast (A), degraded cytoplasm (DC), detached cytoplasm from cell wall (SCW).
(JPG)

## References

[1] Ryan G. Resistance of common groundsel to simazine and atrazine. Weed Sci. 1970: 614–6.

[2] Heap I, editor International survey of herbicide-resistant weeds. Western Society of Weed Science (USA); 1997.

[3] Gressel J, Segel L. Interrelating factors controlling the rate of appearance of resistance: the outlook for the future Herbicide Resistance in Plants New York: J Wiley 1982;215:245.

[4] Duke SO. Why have no new herbicide modes of action appeared in recent years? Pest Manag Sci. 2012;68(4): 505–12. 10.1002/ps.2333 22190296

[5] Araniti F, Mancuso R, Lupini A, Giofrè SV, Sunseri F, Gabriele B, et al Phytotoxic potential and biological activity of three synthetic coumarin derivatives as new natural-like herbicides. Molecules. 2015;20(10): 17883–902. 10.3390/molecules201017883 26426002 PMC6331834

[6] Kessler A, Baldwin IT. Defensive function of herbivore-induced plant volatile emissions in nature. Science. 2001;291(5511): 2141–4. 11251117 10.1126/science.291.5511.2141

[7] Gershenzon J, Dudareva N. The function of terpene natural products in the natural world. Nat Chem Biol. 2007;3(7): 408–14. 17576428 10.1038/nchembio.2007.5

[8] Dudareva N, Negre F, Nagegowda DA, Orlova I. Plant volatiles: recent advances and future perspectives. CRC Cr Rev Plant Sci. 2006;25(5): 417–40.

[9] Vuorinen T, Reddy G, Nerg A-M, Holopainen JK. Monoterpene and herbivore-induced emissions from cabbage plants grown at elevated atmospheric CO_2_ concentration. Atmos Environ. 2004;38(5): 675–82.

[10] Kunert G, Reinhold C, Gershenzon J. Constitutive emission of the aphid alarm pheromone,(E)-β-farnesene, from plants does not serve as a direct defense against aphids. BMC eEcol. 2010;10(1): 1. 10.1186/1472-6785-10-23 PMC3002888 21092302

[11] Fernandes A, Duffield R, Wheeler J, LaBerge W. Chemistry of the Dufour's gland secretions of North American andrenid bees (Hymenoptera: Andrenidae). J Chem Ecol. 1981;7(2): 453–63. 10.1007/BF00995768 24420491

[12] Ali MF, Morgan ED, Attygalle AB, Billen JP. Comparison of Dufour gland secretions of two species of *Leptothorax* ants (Hymenoptera: Formicidae). Zeitschrift für Naturforschung C. 1987;42(7–8): 955–60.

[13] Jackson BD, Morgan ED, Billen J. Contents of the pygidial gland of the ant *Myrmecia nigriceps*. Naturwissenschaften. 1990;77(4): 187–8.

[14] Honda K. GC-MS and 13 C-NMR studies on the biosynthesis of terpenoid defensive secretions by the larvae of papilionid butterflies (*Luehdorfia* and *Papilio*). Insect Biochem. 1990;20(3): 245–50.

[15] Araniti F, Graña E, Reigosa MJ, Sánchez-Moreiras AM, Abenavoli MR. Individual and joint activity of terpenoids, isolated from *Calamintha nepeta* extract, on *Arabidopsis thaliana*. Nat Prod Res. 2013;27(24): 2297–303. 10.1080/14786419.2013.827193 23972283

[16] Hashimoto T. Molecular genetic analysis of left–right handedness in plants. Philosophical transactions of the royal society of London B: Biological Sciences. 2002;357(1422): 799–808. 12079675 10.1098/rstb.2002.1088 PMC1692985

[17] Sedbrook JC, Ehrhardt DW, Fisher SE, Scheible W-R, Somerville CR. The *Arabidopsis* SKU6/SPIRAL1 gene encodes a plus end–localized microtubule-interacting protein involved in directional cell expansion. Plant Cell. 2004;16(6): 1506–20. 15155883 10.1105/tpc.020644 PMC490042

[18] Ishida T, Thitamadee S, Hashimoto T. Twisted growth and organization of cortical microtubules. J Plant Res. 2007;120(1):61–70. 17061141 10.1007/s10265-006-0039-y

[19] Thitamadee S, Tuchihara K, Hashimoto T. Microtubule basis for left-handed helical growth in *Arabidopsis*. Nature. 2002;417(6885): 193–6. 12000963 10.1038/417193a

[20] Furutani I, Watanabe Y, Prieto R, Masukawa M, Suzuki K, Naoi K, et al The SPIRAL genes are required for directional control of cell elongation in *Arabidopsis thaliana*. Development. 2000;127(20): 4443–53. 11003843 10.1242/dev.127.20.4443

[21] Nakamura M, Naoi K, Shoji T, Hashimoto T. Low concentrations of propyzamide and oryzalin alter microtubule dynamics in *Arabidopsis* epidermal cells. Plant Cell Physiol. 2004;45(9): 1330–4. 15509858 10.1093/pcp/pch300

[22] Chaimovitsh D, Altshuler O, Belausov E, Abu-Abied M, Rubin B, Sadot E, et al The relative effect of citral on mitotic microtubules in wheat roots and BY2 cells. Plant Biol. 2012;14(2): 354–64. 10.1111/j.1438-8677.2011.00511.x 22039835

[23] Morejohn L, Bureau T, Mole-Bajer J, Bajer A, Fosket D. Oryzalin, a dinitroaniline herbicide, binds to plant tubulin and inhibits microtubule polymerization *in vitro*. Planta. 1987;172(2): 252–64. 10.1007/BF00394595 24225878

[24] Hoffman J, Vaughn K. Mitotic disrupter herbicides act by a single mechanism but vary in efficacy. Protoplasma. 1994;179(1–2): 16–25.

[25] Deysson G. Microtubules and antimitotic substances: North-Holland publ; 1975.

[26] Vaughn KC, Vaughan MA, editors. Mitotic disrupters from higher plants. Effects on plant cells. ACS Symposium series-American Chemical Society (USA); 1988.

[27] Cortese F, Bhattacharyya B, Wolff J. Podophyllotoxin as a probe for the colchicine binding site of tubulin. J Biol Chem. 1977;252(4): 1134–40. 14143

[28] Hillmann G, Ruthmann A. Effect of mitotic inhibitors on the ultrastructure of root meristem cells. Planta. 1982;155(2): 124–32. 10.1007/BF00392542 24271665

[29] Oliva A, Moraes R, Watson S, Duke S, Dayan F. Aryltetralin lignans inhibit plant growth by affecting the formation of mitotic microtubular organizing centers. Pestic Biochem Phys. 2002;72(1): 45–54.

[30] Graña E, Sotelo T, Díaz-Tielas C, Araniti F, Krasuska U, Bogatek R, et al Citral induces auxin and ethylene-mediated malformations and arrests cell division in *Arabidopsis thaliana* roots. J Chem Ecol. 2013;39(2): 271–82. 10.1007/s10886-013-0250-y 23389342

[31] Gniazdowska A, Krasuska U, Andrzejczak O, Soltys D. Allelopathic compounds as oxidative stress agents: YES or NO Reactive Oxygen and Nitrogen Species Signaling and Communication in Plants: Springer; 2015 p. 155–76.

[32] Yu M, Lamattina L, Spoel SH, Loake GJ. Nitric oxide function in plant biology: a redox cue in deconvolution. New Phytol. 2014;202(4): 1142–56. 10.1111/nph.12739 24611485

[33] Potters G, Pasternak TP, Guisez Y, Jansen MA. Different stresses, similar morphogenic responses: integrating a plethora of pathways. Plant Cell Environ. 2009;32(2): 158–69. 10.1111/j.1365-3040.2008.01908.x 19021890

[34] Potters G, Pasternak TP, Guisez Y, Palme KJ, Jansen MA. Stress-induced morphogenic responses: growing out of trouble? Trends Plant Sci. 2007;12(3): 98–105. 17287141 10.1016/j.tplants.2007.01.004

[35] Schopfer P. Hydroxyl radical-induced cell-wall loosening *in vitro* and in vivo: implications for the control of elongation growth. Plant J. 2001;28(6): 679–88. 11851914 10.1046/j.1365-313x.2001.01187.x

[36] Joo JH, Bae YS, Lee JS. Role of auxin-induced reactive oxygen species in root gravitropism. Plant Phys. 2001;126(3): 1055–60. 10.1104/pp.126.3.1055 PMC116462 11457956

[37] Araniti F, Sánchez-Moreiras AM, Graña E, Reigosa MJ, Abenavoli MR. Terpenoid *trans*-caryophyllene inhibits weed germination and induces plant water status alteration and oxidative damage in adult *Arabidopsis*. Plant Biol. 2016 *In press*. 10.1111/plb.12471 27173056

[38] Holzinger A, Wasteneys G, Lütz C. Investigating cytoskeletal function in chloroplast protrusion formation in the arctic-alpine plant *Oxyria digyna*. *Plant Biol*. 2007;9(3): 400–10. 17236103 10.1055/s-2006-924727

[39] Holzinger A, Kawamura E, Wasteneys GO. Strategies for imaging microtubules in plant cells. Cytoskeleton Methods and Protocols. 2010: 243–62. 10.1007/978-1-60761-376-3_13 19768434

[40] Idris EE, Bochow H, Ross H, Borriss R. Use of Bacillus subtilis as biocontrol agent. VI. Phytohormonelike action of culture filtrates prepared from plant growth-promoting *Bacillus amyloliquefaciens* FZB24, FZB42, FZB45 and *Bacillus subtilis* FZB37/Nutzung von *Bacillus subtilis* als Mittel für den biologischen Pflanzenschutz. VI. Phytohormonartige Wirkung von Kulturfiltraten von pflanzenwachstumsfördernden Bacillus amyloliquefaciens FZB24, FZB42, FZB45 und *Bacillus subtilis* FZB37. Zeitschrift für Pflanzenkrankheiten und Pflanzenschutz/ J Plant Dis Prot. 2004: 583–97.

[41] van Acker FA, Schouten O, Haenen GR, van der Vijgh WJ, Bast A. Flavonoids can replace α-tocopherol as an antioxidant. FEBS letters. 2000;473(2): 145–8. 10812062 10.1016/s0014-5793(00)01517-9

[42] Reichheld J P, Vernoux T, Lardon F, Van Montagu M, Inzé D. Specific checkpoint regulate plant cell cycle progression in response to oxidative stress. Plant J. 1999; 17: 647–656

[43] Schwarzerová K, Zelenková S, Nick P, Opatrný Z. Aluminum-induced rapid changes in the microtubular cytoskeleton of tobacco cell lines. Plant Cell Physiol. 2002; 43: 207–216. 11867700 10.1093/pcp/pcf028

[44] Kovtun Y, Chiu W L, Tena G, Sheen J. Functional analysis of oxidative stress activated mitogen-activated protein kinase cascade in plants. Proc. Natl. Acad. Sci. U. S. A. 2000; 97: 2940–2945. 10717008 10.1073/pnas.97.6.2940 PMC16034

[45] Uddin M, Li X, Won O, Park S, Pyon J. Herbicidal activity of phenolic compounds from hairy root cultures of *Fagopyrum tataricum*. Weed Res. 2012;52(1): 25–33.

[46] Galindo JC, Hernández A, Dayan FE, Tellez MR, Macıías FA, Paul RN, et al Dehydrozaluzanin C, a natural sesquiterpenolide, causes rapid plasma membrane leakage. Phytochemistry. 1999;52(5): 805–13.

[47] Alonso JM, Stepanova AN, Solano R, Wisman E, Ferrari S, Ausubel FM, et al Five components of the ethylene-response pathway identified in a screen for weak ethylene-insensitive mutants in *Arabidopsis*. P Natl Acad Sci. 2003;100(5): 2992–7. 10.1073/pnas.0438070100 PMC151454 12606727

[48] Casimiro I, Marchant A, Bhalerao RP, Beeckman T, Dhooge S, Swarup R, et al Auxin transport promotes *Arabidopsis* lateral root initiation. Plant Cell. 2001;13(4): 843–52. 11283340 10.1105/tpc.13.4.843 PMC135543

[49] Rahman A, Hosokawa S, Oono Y, Amakawa T, Goto N, Tsurumi S. Auxin and ethylene response interactions during *Arabidopsis* root hair development dissected by auxin influx modulators. Plant Phys. 2002;130(4): 1908–17. 10.1104/pp.010546 PMC166701 12481073

[50] Swarup R, Parry G, Graham N, Allen T, Bennett M. Auxin cross-talk: integration of signalling pathways to control plant development Auxin Molecular Biology: Springer; 2002 p. 411–26. 10.1007/978-94-010-0377-3_12 12036264

[51] Lewis DR, Negi S, Sukumar P, Muday GK. Ethylene inhibits lateral root development, increases IAA transport and expression of PIN3 and PIN7 auxin efflux carriers. Development. 2011;138(16): 3485–95. 10.1242/dev.065102 21771812

[52] Li X, Gruber MY, Hegedus DD, Lydiate DJ, Gao M-J. Effects of a coumarin derivative, 4-methylumbelliferone, on seed germination and seedling establishment in *Arabidopsis*. J Chem Ecol. 2011;37(8): 880–90. 10.1007/s10886-011-9987-3 21713565

[53] Lupini A, Araniti F, Sunseri F, Abenavoli MR. Coumarin interacts with auxin polar transport to modify root system architecture in *Arabidopsis thaliana*. Plant Growth Regul. 2014;74(1): 23–31.

[54] Ivanchenko MG, Muday GK, Dubrovsky JG. Ethylene–auxin interactions regulate lateral root initiation and emergence in *Arabidopsis thaliana*. Plant J. 2008;55(2): 335–47. 10.1111/j.1365-313X.2008.03528.x 18435826

[55] Graña E, Araniti F, Abenavoli MR, Reigosa MJ, Sánchez-Moreiras AM, editors. Effects of farnesene on root morphology of *Arabidopsis thaliana* Heynh Plant Biology Congress; 2012; Freiburg p. 768.

[56] Grossmann K. A role for cyanide, derived from ethylene biosynthesis, in the development of stress symptoms. Physiol Plantarum. 1996;97(4): 772–5.

[57] Tittle FL, Goudey JS, Spencer MS. Effect of 2, 4-dichlorophenoxyacetic acid on endogenous cyanide, *β*-cyanoalanine synthase activity, and ethylene evolution in seedlings of soybean and barley. Plant Physiol. 1990;94(3): 1143–8. 16667809 10.1104/pp.94.3.1143 PMC1077354

[58] Gniazdowska A, Krasuska U, Bogatek R. Dormancy removal in apple embryos by nitric oxide or cyanide involves modifications in ethylene biosynthetic pathway. Planta. 2010;232(6): 1397–407. 10.1007/s00425-010-1262-2 20830596

[59] Correa-Aragunde N, Graziano M, Lamattina L. Nitric oxide plays a central role in determining lateral root development in tomato. Planta. 2004;218(6): 900–5. 14716561 10.1007/s00425-003-1172-7

[60] Tsukagoshi H, Busch W, Benfey PN. Transcriptional regulation of ROS controls transition from proliferation to differentiation in the root. Cell. 2010;143(4): 606–16. 10.1016/j.cell.2010.10.020 21074051

[61] Dunand C, Crèvecoeur M, Penel C. Distribution of superoxide and hydrogen peroxide in *Arabidopsis* root and their influence on root development: possible interaction with peroxidases. New Phytol. 2007;174(2): 332–41. 17388896 10.1111/j.1469-8137.2007.01995.x

[62] Causin HF, Roqueiro G, Petrillo E, Láinez V, Pena LB, Marchetti CF, et al The control of root growth by reactive oxygen species in *Salix nigra* Marsh. seedlings. Plant Sci. 2012;183: 197–205. 10.1016/j.plantsci.2011.08.012 22195594

[63] Guan Y, Lin H, Ma L, Yang Y, Hu X. Nitric oxide and hydrogen peroxide are important signals mediating the allelopathic response of *Arabidopsis* to *p*-hydroxybenzoic acid. Physiol Plantarum. 2014;152(2): 275–85. 10.1111/ppl.12164 24502504

[64] Soltys D, Rudzińska-Langwald A, Kurek W, Gniazdowska A, Sliwinska E, Bogatek R. Cyanamide mode of action during inhibition of onion (*Allium cepa* L.) root growth involves disturbances in cell division and cytoskeleton formation. Planta. 2011;234(3): 609–21. 10.1007/s00425-011-1429-5 21573814 PMC3162148

[65] Soltys D, Rudzińska-Langwald A, Kurek W, Szajko K, Sliwinska E, Bogatek R, et al Phytotoxic cyanamide affects maize (*Zea mays*) root growth and root tip function: from structure to gene expression. J Plant Physiol. 2014;171(8): 565–75. 10.1016/j.jplph.2014.01.004 24709147

[66] Romero-Puertas M, McCarthy I, Gómez M, Sandalio L, Corpas F, Del Rio L, et al Reactive oxygen species-mediated enzymatic systems involved in the oxidative action of 2, 4-dichlorophenoxyacetic acid. Plant Cell Environ. 2004;27(9): 1135–48.

[67] Kasprowicz A, Szuba A, Volkmann D, Baluška F, Wojtaszek P. Nitric oxide modulates dynamic actin cytoskeleton and vesicle trafficking in a cell type-specific manner in root apices. J Exp Bot. 2009;60(6): 1605–17. 10.1093/jxb/erp033 19261922 PMC2671617

[68] Fernández-Marcos M, Sanz L, Lewis DR, Muday GK, Lorenzo O. Nitric oxide causes root apical meristem defects and growth inhibition while reducing PIN-FORMED 1 (PIN1)-dependent acropetal auxin transport. P Natl A Sci. 2011;108(45): 18506–11. 10.1073/pnas.1108644108 PMC3215072 22021439

[69] Walne PL, Haber AH, Triplett L. Ultrastructure of auxin-induced tumors of the coleorhiza-epiblast of wheat. Am J Bot. 1975: 58–66. 30139107 10.1002/j.1537-2197.1975.tb12338.x

[70] Soltys D, Rudzińska-Langwald A, Gniazdowska A, Wiśniewska A, Bogatek R. Inhibition of tomato (*Solanum lycopersicum* L.) root growth by cyanamide is due to altered cell division, phytohormone balance and expansin gene expression. Planta. 2012;236(5): 1629–38. 10.1007/s00425-012-1722-y 22847024 PMC3481057

[71] Lee JS, Chang W-K, Evans ML. Effects of ethylene on the kinetics of curvature and auxin redistribution in gravistimulated roots of *Zea mays*. Plant Physiol. 1990;94(4): 1770–5. 11537475 10.1104/pp.94.4.1770 PMC1077451

[72] Muday GK, Rahman A, Binder BM. Auxin and ethylene: collaborators or competitors? Trends Plant Sci. 2012;17(4): 181–95. 10.1016/j.tplants.2012.02.001 22406007

[73] Ellis JR, Taylor R, Hussey PJ. Molecular modeling indicates that two chemically distinct classes of anti-mitotic herbicide bind to the same receptor site (s). Plant Physiol. 1994;105(1): 15–8. 12232182 10.1104/pp.105.1.15 PMC159324

[74] Fisher DD, Cyr RJ. Extending the microtubule/microfibril paradigm cellulose synthesis is required for normal cortical microtubule alignment in elongating cells. Plant Physiol. 1998;116(3): 1043–51. 9501137 10.1104/pp.116.3.1043 PMC35074

[75] Morejohn L. The molecular pharmacology of plant tubulin and microtubules The cytoskeletal basis of plant growth and form. 1991: 29–43.

[76] Green P, Selker J. Mutual alignments of cell walls, cellulose, and cytoskeletons: their role in meristems The Cytoskeletal Basis of Plant Growth and Form Academic Press, San Diego, CA 1991: 303–22.

[77] Vaughn KC, Marks MD, Weeks DP. A dinitroaniline-resistant mutant of *Eleusine indica* exhibits cross-resistance and supersensitivity to antimicrotubule herbicides and drugs. Plant Physiol. 1987;83(4): 956–64. 16665371 10.1104/pp.83.4.956 PMC1056482

[78] Sugimoto K, Himmelspach R, Williamson RE, Wasteneys GO. Mutation or drug-dependent microtubule disruption causes radial swelling without altering parallel cellulose microfibril deposition in *Arabidopsis* root cells. Plant Cell. 2003;15(6): 1414–29. 12782733 10.1105/tpc.011593 PMC156376

[79] Baskin TI, Beemster GT, Judy-March JE, Marga F. Disorganization of cortical microtubules stimulates tangential expansion and reduces the uniformity of cellulose microfibril alignment among cells in the root of *Arabidopsis*. Plant Physiol. 2004;135(4): 2279–90. 15299138 10.1104/pp.104.040493 PMC520797

[80] Cleary A, Hardham A. Depolymerization of microtubule arrays in root tip cells by oryzalin and their recovery with modified nucleation patterns. Canadian J Bot. 1988;66(12): 2353–66.

