# Supplementary figures and images for: Loss of Gravitropism in Farnesene-Treated Arabidopsis Is Due to Microtubule Malformations Related to Hormonal and ROS Unbalance
Source: PLoS One. 2016 Aug 4;11(8):e0160202. doi: 10.1371/journal.pone.0160202 (PMC4974009; doi:10.1371/journal.pone.0160202)

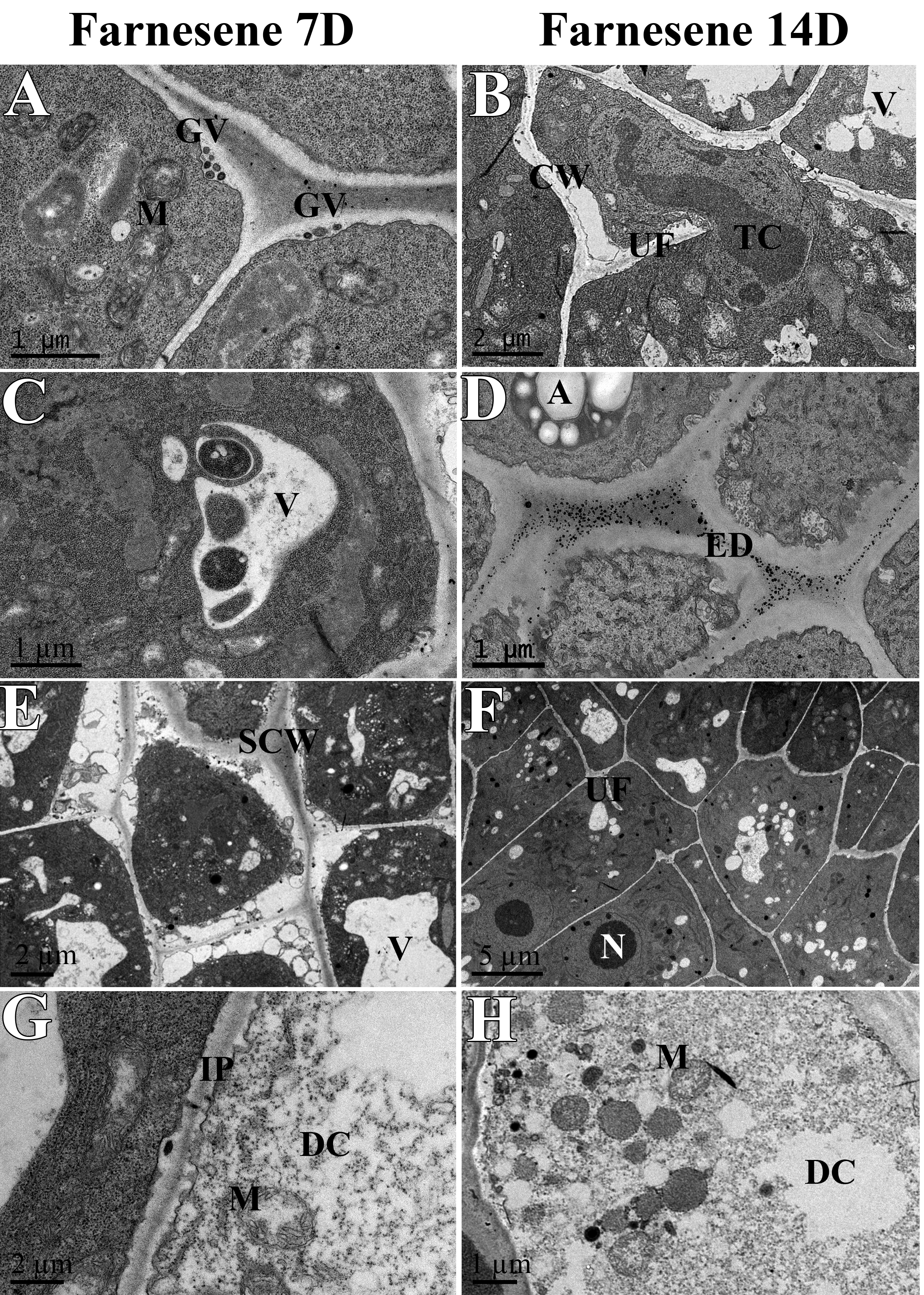

Supplement: S2 Fig — TEM microphotographs of the apical meristem of 7 days (A, C, E) and 14 days (B, D, F) treated Arabidopsis roots.: A) Signs of active exocytosis and mitochondria with irregular shape and translucent stroma; B) Tetranucleated cell showing incomplete cell wall; C) vacuoles autophagy activity; D) Electro-dense deposit on cell wall); E) Degradation of the cytoplasm in the elongation zone and plasmatic membrane detached from the cell wall; F) Protodermal cells with incomplete phragmoplast, abnormal shape and swollen cell walls; G) and H) Degraded cytoplasm and mitochondria with irregular morphology and translucent stroma. Nucleus (N), vacuole (V), cell wall (CW), mitochondria (M), incomplete plasmodesmata (IP), Golgi vesicles (GV), uncompleted phragmoplast (UF), tetranucleated cell (TC), electro-dense deposits (ED), amyloplast (A), degraded cytoplasm (DC), detached cytoplasm from cell wall (SCW). (JPG) [file pone.0160202.s002.jpg]
